# Targeting blood brain barrier—Remote ischemic conditioning alleviates cognitive impairment in female APP/PS1 rats

**DOI:** 10.1111/cns.14613

**Published:** 2024-02-20

**Authors:** Yuxuan Ma, Wuxiang Sun, Jing Bai, Fujia Gao, Haoran Ma, Huiyu Liu, Jiewei Hu, Chao Xu, Xin Zhang, Zixuan Liu, Tao Yuan, Chenxu Sun, Yuanyuan Huang, Ruimin Wang

**Affiliations:** ^1^ International Science & Technology Cooperation Base of Geriatric School of Public Health of North China University of Science and Technology Tangshan Hebei China; ^2^ School of Basic Medical Science North China University of Science and Technology Tangshan Hebei China

**Keywords:** Alzheimer's disease, blood–brain barrier, cognitive impairment, neuron vascular unit, remote ischemic conditioning

## Abstract

**Aims:**

Alzheimer's disease (AD) is a significant global health concern, and it is crucial that we find effective methods to prevent or slow down AD progression. Recent studies have highlighted the essential role of blood vessels in clearing Aβ, a protein that contributes to AD. Scientists are exploring blood biomarkers as a potential tool for future AD diagnosis. One promising method that may help prevent AD is remote ischemic conditioning (RIC). RIC involves using sub‐lethal ischemic–reperfusion cycles on limbs. However, a comprehensive understanding of how RIC can prevent AD and its long‐term effectiveness is still lacking. Further research is essential to fully comprehend the potential benefits of RIC in preventing AD.

**Methods:**

Female wild‐type (WT) and APP/PS1 transgenic rats, aged 12 months, underwent ovariectomy and were subsequently assigned to WT, APP/PS1, and APP/PS1 + RIC groups. RIC was conducted five times a week for 4 weeks. The rats' depressive and cognitive behaviors were evaluated using force swimming, open‐field tests, novel objective recognition, elevated plus maze, and Barnes maze tests. Evaluation of the neurovascular unit (NVU), synapses, vasculature, astrocytes, and microglia was conducted using immunofluorescence staining (IF), Western blot (WB), and transmission electron microscopy (TEM). Additionally, the cerebro‐vasculature was examined using micro‐CT, and cerebral blood flow (CBF) was measured using Speckle Doppler. Blood–brain barrier (BBB) permeability was determined by measuring the Evans blue leakage. Finally, Aβ levels in the rat frontal cortex were measured using WB, ELISA, or IF staining.

**Results:**

RIC enhanced memory‐related protein expression and rescued depressive‐like behavior and cognitive decline in APP/PS1 transgenic rats. Additionally, the intervention protected NVU in the rat frontal cortex, as evidenced by (1) increased expression of TJ (tight junction) proteins, pericyte marker PDGFRβ, and glucose transporter 1 (GLUT1), as well as decreased VCAM1; (2) mitigation of ultrastructure impairment in neuron, cerebral vascular, and astrocyte; (3) upregulation of A2 astrocyte phenotype markers and downregulation of A1 phenotype markers, indicating a shift toward a healthier phenotype. Correspondingly, RIC intervention alleviated neuroinflammation, as evidenced by the decreased Iba1 level, a microglia marker. Meanwhile, RIC intervention elevated CBF in frontal cortex of the rats. Notably, RIC intervention effectively suppressed Aβ toxicity, as demonstrated by the enhancement of α‐secretase and attenuation of β‐secretase (BACE1) and γ‐ secretase and Aβ1‐42 and Aβ1‐40 levels as well.

**Conclusion:**

Chronic RIC intervention exerts vascular and neuroprotective roles, suggesting that RIC could be a promising therapeutic strategy targeting the BBB and NVU during AD development.

## Graphical abstract

This study systematically elucidated the positive effects of RIC on middle‐aged APP/PS1 rats. The findings highlight the importance of prioritizing the cerebrovascular system as a crucial component for preventing AD pathologies, such as improved CBF, BBB integrity, astrocyte remodeling, and reduced Aβ peptide, while also preserving cognition.

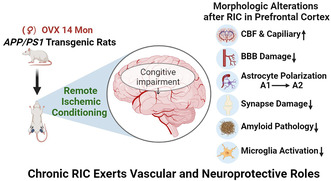

## 1 INTRODUCTION

As of 2022, there were over 800 million people aged 65 or older in the world, accounting for 10% of the global population. These individuals are at a higher risk of developing mild cognitive impairment (MCI), with approximately one‐third of them progressing to dementia due to Alzheimer's disease (AD) within 5 years of MCI onset.^1^, ^2^ An estimated one in nine people age 65 and older has AD, and the total payments for healthcare, long‐term care, and hospice services will be $321 billion in 2022.^3^ Pathologically, AD is characterized by the aggregation and accumulation of amyloid plaques in the brain parenchyma, abnormally phosphorylated tau in neurons (neurofibrillary tangles), and associated loss of synapses.^4^, ^5^ In addition to these classic pathological hallmarks, cerebrovascular dysfunction during AD progression has also been well‐documented and received considerable attention lately. With respect to diagnosis of AD, it has been suggested that blood biomarkers may hold the most promise for future diagnostic approaches and are more scalable than cerebrospinal fluid (CSF) and brain imaging markers.^6^

The brain is a unique site in the body that is immune‐privileged due to the presence of the blood–brain barrier (BBB). The BBB is composed of three cellular elements: endothelial cells (ECs), astrocyte end‐feet, and pericytes, which hold together tight junction proteins (TJs), such as zonula occludin protein 1 *(*ZO‐1), claudins, and occludin. Thus, the BBB constitutes a diffusion barrier between blood and brain parenchyma, providing a large surface area and an important mediator for the influx of what is needed and the efflux of what could be harmful across the cerebral vasculature.^7^, ^8^ Indeed, the BBB plays an important role in the removal of amyloid‐β (Aβ) from the brain both in humans and in animal models.^9^, ^10^ Further work has shown that BBB disruption, degeneration of TJ proteins, and loss of pericytes exacerbate brain aging^11^ and contribute to AD pathogenesis in *APP* ^sw/0^ mice^12^ and individuals with AD.^13^ Conversely, the toxic effects of Aβ are thought to negatively impact cerebrovascular function by promoting oxidative stress, chronic inflammation, and impairing endothelial structure and function.^14^ Soluble Aβ oligomers can also facilitate astrocyte hyperactivity, which in turn affects neighboring hippocampal CA1 neurons, triggering an increase in glutamatergic spontaneous activity.^15^

To date, the U.S. Food and Drug Administration (FDA) has approved six drugs for treating AD: donepezil, rivastigmine, galantamine, memantine, and memantine combined with donepezil, along with aducanumab.^16^ Although the first five drugs can temporarily improve AD symptoms, they do not modify the underlying brain changes of AD and may cause mild side effects, such as headache and nausea.^3^ In contrast, aducanumab, which was approved by the FDA in June 2021,^17^ works by reducing Aβ plaques in the brain and addresses the biology of AD. However, it does not cure AD and may not be suitable for all individuals living with AD due to the most common adverse events, such as amyloid‐related imaging abnormalities (ARIA)‐edema and ARIA‐headache.^18^, ^19^ With the latest understanding of new pathological causes of AD, more and more therapeutic targets for AD will be unrevealed. Nevertheless, the side effects of multi‐target medications for the elderly cannot be ignored. Therefore, parallel avenues of research to develop non‐pharmaceutical intervention modalities are much needed.

One potential non‐pharmacological intervention being investigated is remote limb ischemic preconditioning, herein termed remote ischemic conditioning (RIC). RIC is a noninvasive approach that triggers the body's endogenous protective capabilities by several cycles of non‐lethal ischemia–reperfusion on limbs.^20^ RIC has been extensively used as an auxiliary treatment for cardio‐ and cerebrovascular disorders such as coronary heart disease and stroke.^21^, ^22^, ^23^ Recent studies have demonstrated that RIC can enhance skin microcirculation in normal adults^24^ and increase muscle strength in young and old individuals.^25^ Additionally, RIC has been reported to enhance motor learning ability in middle‐aged and elderly people,^26^ improve vascular endothelial function and brain blood supply insufficiency,^27^ and protect against stroke by reducing inflammation.^28^ Notably, RIC can regulate immune cells to protect the ischemic brain.^29^, ^30^, ^31^ RIC performed in healthy volunteers confirmed that serum monocytes and cytokines isolated from the volunteers can synergistically promote cardiac microvascular regeneration and up‐regulation of angiogenesis‐related factors.^32^ Hess et al have proposed that RIC is a “protective agent” for the heart and central nervous system, which is equivalent to exercise, and has the most medical transformation value, particularly for people who cannot or do not want to exercise.^33^

Building on these findings, we conducted the current study to (1) clarify whether chronic RIC conducted on double hind limbs of *APP/PS1* rats could preserve vascular integrity, including TJs, ECs, pericytes, astrocytes, and microglia and (2) confirm whether RIC can prevent synaptic loss and cognitive dysfunction in *APP/PS1* rats. To accomplish these aims, we generated *APP/PS1* transgenic rats in the F344 background and 12‐month‐old ovariectomized (OVX) female rats were used for RIC intervention. This design was made based on the facts that (1) 12‐month‐old rats share similar characteristics with middle‐aged humans who are at a higher risk of depression and MCI; (2) women over 65 years of age are more prone to AD with two–three times than men of the same age,^34^ (3) and women who undergo premature surgical menopause (bilateral oophorectomy) are at an increased risk of cognitive decline, dementia, and mortality from neurological disorders.^35^, ^36^, ^37^ We hypothesized that noninvasive RIC intervention could ameliorate cognitive dysfunction in *APP/PS1* rats by safeguarding the NVU. Our research may provide a new perspective on the prevention and treatment of AD.

## 2 MATERIALS AND METHODS

### 2.1 Materials

#### 2.1.1 Antibodies and animals

The primary antibodies used for immunofluorescence staining or Western blot analysis in this study are listed and described in Table 1. *APP/PS1* double transgenic F344 (Tg‐F344‐AD) rats were generated on a Fischer 344 background by Beijing Weishang Lide Biotechnology Co., Ltd (Code No: FW20181025‐V1). Two human genes: Swedish *APP* (*APPsw*) and presenilin‐1 (*PS1*△*E9*) driven by the pCAG promoter were efficiently integrated between two ITR (reverse terminal repeat) sites of PiggyBac and co‐injected into rat pronuclei. PiggyBac has a more precise “cut and paste” mechanism,^38^, ^39^, ^40^, ^41^, ^42^ higher transposition efficiency, and larger cargo capacity.^43^, ^44^, ^45^ Transgene integration was confirmed by genotyping, and expression levels were evaluated by ELISA analysis of brain homogenates.

**TABLE 1:** Antibody information.

Antibodies	IF	WB	Companies	Species	RRID	Cat No.
GAPDH	/	1:12,000	Proteintech	M	AB_2107436	Cat N0.60004‐1‐lg
C3b/c	1:200	1:1000	Proteintech	R	AB_2198052	Cat N0.21337‐1‐AP
ZO‐1	1:100	/	Santa Cruz	R	AB_2205514	Sc‐10,804
Occludin	1:50	/	Santa Cruz	G	AB_653537	Sc‐8144
RECA1	1:100	/	Santa Cruz	M	AB_1128987	Sc‐71,958
PDGFRβ	1:100	1:500	HUABIO	R	AB_2728816	ET1605‐20
VCAM1	1:1000	1:1000	Abcam	R	AB_2721053	Ab134047
GFAP	1:1000	1:1000	Abcam	G	AB_880202	Ab53554
GLUT1	1:1000	1:800	Immunoway	R	AB_10986893	YT1928
BACE1	1:1000	1:1200	Abcam	R	AB_10861218	Ab183612
PSD95	/	1:800	Abcam	R	AB_444362	Ab18258
Synaptotagmin	/	1:200	Abcam	M	AB_299799	Ab13259
Syntaxin	/	1:150	Abcam	M	AB_303654	Ab3265
VAMP2	/	1:1000	Abcam	R	AB_2721005	Ab181869
BDNF	/	1:500	ARIGO	R	AB_2039756	ARG56653
Vimentin	/	1:40,000	Proteintech	M	AB_2881439	60,330
PTX3	1:100	1:500	Abclonal	R	AB_2759517	A12670
S100A10	/	1:500	Affinity bioscience	R	AB_2837666	AF5180
Aβ1‐16(6‐E10)	1:40,000	/	BioLegend	M	AB_2734556	805,707
4G8	1:1000	/	BioLegend	M	AB_10175152	Catalog# SIG‐39200

### 2.2 Methods

#### 2.2.1 Experimental design and remote ischemic conditioning (RIC) intervention

**FIGURE 1:**
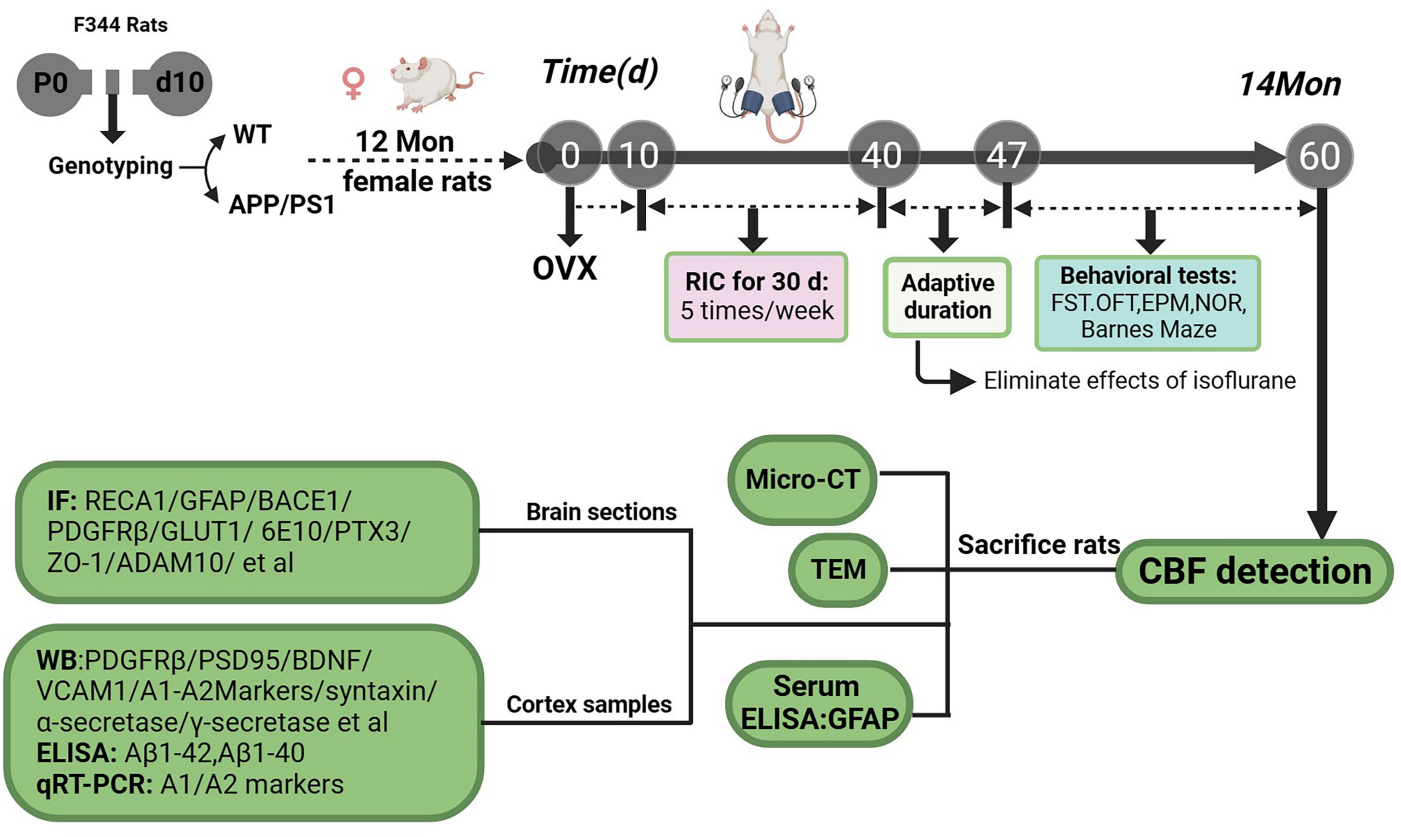
Schematic diagram of the experimental design. Animals were subjected to genotyping on postnatal day 10 (P10), and then female rats were separated into two groups at one Mon: WT and APP/PS1. We selected 12‐month‐old rats, who underwent bilateral ovariectomy (day 0), and 10 days later, half of APP/PS1 rats received RIC intervention for a period of 1 month, five times a week. To eliminate potential side effects of isoflurane and RIC, all rats were allowed to acclimatize for 1 week, and then underwent behavioral tests, and CBF was measured. All rats were sacrificed 60 days after OVX (14 months old). Brain tissue, cortex tissues, and serum samples were collected for Micro‐CT, TEM and GFAP level measurement, respectively. Brain sections and cortex samples were used for immunofluorescence staining and Western blot analysis.

Animals were raised in an environment with an ambient temperature of 22–24°C and moisture of 50–60% under a 12‐h light/dark cycle. All experiments were conducted following the National Natural Science Foundation of China for the Care and Use of Laboratory Animals and were approved by the Animal Care Committee of North China University of Science and Technology. A schematic diagram of the experimental design is shown in Figure 1. Genotyping was performed on the rats at postnatal day 10, and at 1 month, they were divided into two groups: WT and APP/PS1. For the experiments, 12‐month‐old female rats (WT and APP/PS1) were utilized, and all rats underwent bilateral ovariectomy (OVX) on day 0. The APP/PS1 rats were randomly assigned to RIC (+RIC) and non‐RIC (‐RIC) intervention subgroups. Ten days later after OVX, RIC intervention was conducted for 1 month as previously described with minor modification.^46^ Briefly, the non‐invasive occlusion was achieved using a tailored set of blood pressure monitoring systems consisting of minimal cuffs and a gasbag, 230 ± 10 mmHg × four cycles, 10 min each × 10‐min interval between cycles. In order to eliminate the potential side effects of isoflurane on RIC performance, all rats were allowed adaptive maintenance in the standard environment for 1 week, and then we conducted behavioral tests and measured the CBF. At 60 days after OVX (14‐month age), all rats were humanely sacrificed, and both serum and tissue samples were collected.

#### 2.2.2 Regional cerebral blood flow monitoring

Cerebral blood flow (CBF) in the rat cerebral frontal–parietal cortex was monitored using Laser Speckle Contrast Imaging/LSCI (RWD Life Science CO., LTD, China). Laser speckle contrast imaging was performed as described by Xie et al.^47^ with minor modification. Briefly, the rats were deeply anesthetized under chloral hydrate anesthesia (10%, 0.36 mL/kg i.p.), and the head was fixed on a brain stereotaxic apparatus. The scalp was opened and periosteum removed. Both parietal bones were thinned to translucency under saline cooling (12 × 10 mm) using a high‐speed drill. The rat was then placed under a macro lens (Nikon 60 mm f/2.8 AF‐S; Nikon Inc., Melville, NY, USA), and the aperture was adjusted to match the speckle size to the area of a single pixel in a 12‐bit CCD camera (270XS 11,066; Pixel fly, PCO, Kelheim, Germany). Shutter speed was set to an exposure time of 5 ms, and images were continuously acquired at a rate of 23 frames per second. The imaging field was positioned to include both hemispheres.

#### 2.2.3 Measurement of the cerebral vessel using micro‐computed tomography (Micro‐CT)

Cerebral microvasculature of the rats was observed by micro‐CT according to the previously described protocol with minor modification.^48^, ^49^ Briefly, under anesthesia, rats were perfused transcardially with cold saline and fixed with 80 mL 4% paraformaldehyde. A mixture of Microfil (Flow TEch, Inc., Carver, MA, USA), which is a blue opaque silicone rubber contrast agent, was then slowly injected, prepared into the mixed liquid before use in the volume ratio of MV‐120: thinner: curing agent = 4: 5: 0.45. The adequate perfusion was determined by checking the color of the eyes and tongue of the rats for consistency with that of the perfusion solution. After perfusion, the rats were placed in a 4°C refrigerator overnight to allow the contrast agent to solidify. The brain tissue was removed from the skull and fixed in 4% paraformaldehyde solution for 2–3 days. Finally, the images were obtained by micro‐CT (NEMO‐II Micro CT (NMC‐200), China). To further quantitatively evaluate the vascular unit, the 3D‐micro‐CT photographs were reconstructed and transformed into TIFF format using the Avatar 3 software (NEMO‐II Micro CT (NMC‐200), China), and the processing of the blood vessels further quantitatively analyze using Angio Tool software according to previous description^50^, ^51^, ^52^ AngioTool is a free practical tool that can comprehensively and quantitatively evaluate various blood vessel morphology and spatial parameters, including blood vessel length and density, branch index, and space.^53^ To begin the analysis using the Angio Tool, the TIFF CT image is opened, and the software was used to perform an initial selection of vessels based on estimated parameters, which were identified by a yellow overlay. The vessel diameter was adjusted to remove small background particles and fill holes to refine the selection of vascular regions. Once the analysis was completed, the overlay showed the allatois outline, the skeleton, and the branching points. Analysis data, including those of vessel density, vessel length, and the number of branches, were obtained.

#### 2.2.4 Quantitation of BBB permeability

The Evans blue (EB) extravasation assay was performed to evaluate BBB permeability. WT and age‐matched APP/PS1 rats (3, 8, and 14 Mon) were injected with EB (2% in 0.9% saline; 3 mL/kg, Sigma‐Aldrich) via the femoral vein. Two hours later, the rats were perfused with 0.9% saline under deep anesthesia, and then the brains were cut into slices (2 mm) using a brain slice mold. The brain slices were then weighed and homogenized in N, N‐dimethyl formamide (Sigma‐Aldrich). The samples were centrifuged and collected. The EB concentration in the supernatant was determined with a microplate reader (SpectraMax M5, Molecular Devices) at 620 nm. The amount of EB extravasated from each sample was expressed as micrograms per gram of wet tissue.^54^ Additionally, immunofluorescence staining of IgG was used to evaluate BBB permeability as the same protocol with immunofluorescence staining.

#### 2.2.5 Preparation of cerebral frontal–parietal cortical samples and Western blot analysis

At designed time points, the rats were anesthetized and transcardially perfused with cold saline. The rat brain was quickly decapitated, and the frontal parietal cortex was immediately collected on ice and stored at −80°C. Protein extractions were prepared with lysis buffer (50 mM Tris–HCl, pH 7.4, 150 mM NaCl, 5 mM EDTA, 0.5% NP‐40, 0.1% Triton X‐100, 0.1% SDS, 1 mM PMSF, 1× protein inhibitor mix Complete Mini) and then centrifuged at 10,000 *g* for 10 min at 4°C. Protein concentrations were examined and standardized using a BCA protein assay kit (Pierce, Rockford, USA). The protein sample (20 μg per lane) was separated by 10% SDS‐PAGE and transferred to a polyvinylidene fluoride (PVDF) membrane. The membranes were blocked with 3% BSA for 1 h and then incubated overnight at 4°C with the primary antibodies described in Table 1. Membranes were washed with TBST and incubated with the secondary antibody for 1 h. The signal was visualized by enhanced chemiluminescence (ECL prime; Amersham Biosciences, Piscataway, NJ, USA). The protein levels were quantitatively analyzed using Image Lab software version 5.2.1 (Bio‐Rad). The protein levels were normalized using GAPDH, a loading control.

#### 2.2.6 Enzyme‐linked immunosorbent assay (ELISA)

For serum GFAP measurement, blood samples (0.5 mL) were collected from the right heart ventricle in RNase‐free microfuge tubes and kept on ice. The samples were then centrifuged at 1000 *g* for 15 min at 4°C to obtain supernatants for the experiment. GFAP concentrations in the serum samples were determined by an (ELISA kit Cat# ml730732‐2, Shanghai Enzyme‐linked Biotechnology Co., Ltd., China) as per the manufacturers' instructions. Briefly, 50 μL of the plasma sample or different concentration standard (8, 4, 2, 1, 0.5, and 0.25 pg/mL) was respectively added to a pre‐coated well with GFAP antibody. The same volume of 0.1 M PBS was used for the blank well. Each well, except the blank well, was added with 100 μL of horseradish peroxidase (HRP)‐labeled secondary antibody and incubated at 37°C for 60 min. After washing five times with 450 μL washing buffer, 50 μL substrate A and B were added to each well and incubated at 37°C for 15 min, followed by adding 50 μL of stopping buffer. The optical density was measured at a wavelength of 450 nm.

Aβ1‐42 (ELISA Kit: E‐EL‐R1402c, Elabscience, Wuhan, China) or Aβ1‐40 (ELISA Kit: CSB‐E08299h, CUSABIO, Wuhan, China) contents in the rat cortex were measured according to the manufacturer's instructions. In brief, protein samples of the rat cortex were extracted, and the protein concentration was determined by BCA (P0009; Beyotime, Hangzhou, China) method. The sample (100 μL) at a protein content of 20 μg/μL was added to the microtiter plate that had been pre‐coated with the corresponding antibodies and incubated at 37°C for 90 min. The wells were washed and incubated with a biotinylated antibody working solution at 37°C for 60 min. After washing, samples were incubated with the HRP‐labeled conjugate at 37°C for 30 min, then washed again, and incubated with the substrate solution at 37°C for 15 min. Finally, a stop solution was added, and the optical density was measured at a wavelength of 450 nm.

#### 2.2.7 Quantitative RT‐PCR analysis

To measure mRNA expression of astrocyte A1, A2, and PAN markers, we isolated total RNA from the cortical tissue. The tissue was collected on ice and stored at −80°C immediately. RNA was extracted with the TRIzol reagent (Cat#15596–026; Life Technologies, Carlsbad, CA, USA) following the manufacturer's instructions. The purified RNA was then reverse‐transcribed onto cDNA using the Reverse Transcription System (RR047A; TaKaRa PrimeScript™ RT reagent Kit). Quantitative RT‐PCR analysis of the mRNA levels of target genes was performed using the TB Green Premix Ex Taq kit (RR820A; TAKARA). The real‐time PCR program steps were as follows: 95°C for 5 min, 45 cycles at 95°C for 5 s, 60°C for 5 s, and 72°C for 10 s, followed by 72°C for 1 min. PCR primers used in the study are listed in Table 2. Cycle thresholds (CT) for single reactions were determined using MyiQ software (Bio‐Rad, Hercules, CA, USA), and the target genes were normalized against GAPDH. The 2^−ΔΔCT^ method was used to calculate relative changes in gene expression.

**TABLE 2:** Primers for qPCR.

OligoName	Capital‐sequence (5′ to 3′)‐F	Capital‐sequence (5′ to 3′)‐R
GFAP	AAGTTTGGGAAACTGACACA	TTCCGAGAGGGTACACTAAT
Vimentin	CGTCCACACGCACCTACAG	GGGGGATGAGGAATAGAGGCT
S100A10	TGAAGCAGAAGAAGTAGGC	CGAATTGGAGTTGGATGTTA
Emp1	TCCCTCGTGGTCTTCGTGTTCC	TGGGCGTAGTGGTGAGTGTAGATAG
Sphk1	CGGACGGCAACTCATGTTCTC	GCTCCTGTATTCTCACTCLCCAAGTC
Tm4sf1	CTTCTGTACTGGCTGCTCTGATTGG	CACACTCCGGGCATCGCTAC
Serping1	GGCGGAGAACACCAACCACAAG	TGGCACTCAAGTAGACGGCATTG
TGM1	GGAGCACCTGGACCACGATTCTG	ACAACCTGCCACCCATCAAAGC
PTX3	ATTCTGCTTTGTGCTCTCTGGTCTG	GGGTCCTCGGTGGGATGAAGTC
GBP2	CTCAGCAGCACCTGTCTACAAC	CCACAAAGTTAGCAGAGTCGTCTATCC
FKBP5	AGCCTGGGATATTGGGGTGTCTAC	CCAGCAGAGCCGTAAGCGTATTC
C3D	CCACCACCTCCACCTGTTCTTAATG	GTTCACTCCTTCTCTGGGCTTGG
GAPDH	CAGTATGATTCTACCCACGG	CAGATCCACAACGGATACAT

#### 2.2.8 Immunofluorescence staining and confocal microscopy

Immunofluorescence (IF) staining was performed as described in our previous study.^55^ Briefly, brain sections were washed three times for 10 min in PBS and then blocked in 10% normal donkey serum in 0.1% Triton X‐100 at room temperature for 1 h. The sections were then incubated with primary antibodies (indicated in Table 1) for 48 h on a rocking platform at 4°C. After washing three times with 0.1% Triton X‐100‐PBS, the brain sections were then incubated with appropriate Alexa Fluor donkey anti‐mouse/goat/rabbit secondary antibodies (488/594/647; ThermoFisher) or 488 nm donkey anti‐rat secondary antibody at room temperature for 1 h. Following secondary antibody incubation, the sections were washed in 0.1% Triton X‐100‐PBS for 4 × 10 min, mounted on slides (12–550‐15; Fisher Scientific), and coverslipped with DAPI mounting medium (Lot ZA0210; Vector Laboratories, Inc., Burlingame, CA). Finally, images were captured on a confocal laser microscope (Andor Dragonfly) using 40 × objectives, with the image size set at 1024 × 1024 pixels.

#### 2.2.9 Morphometric measurement of vascular and astrocytic parameters

We stained and visualized the vascular structure with an endothelial marker (RECA 1) and captured confocal Z‐stack images of coronal brain sections spanning the frontal parietal cortex (40 mm thick, 200 mm apart) under a 40 × objective lens by using a confocal microscope. To separate the vascular segments, the acquired fluorescent images were changed into 8 bits, thresholded, noise‐filtered, and binarized using Fiji software (ImageJ, NIH, MD, version 1.52q). To evaluate cerebrovascular BBB disruption, we quantified the vascular density according to a previous report with modification.^56^ Briefly, we segmented the images into three different pixel regions (100–2000, 2001–5000, and 5001–infinity) to correspond with the vascular dimensions. To maximize objectivity, a third researcher was enlisted to conduct the process. A grid was constructed to represent the intensity scales where 100–2000 pixels represents severe vascular damage, 2001–5000 pixels represents mild vascular damage, and 5001–infinity pixels represent normal vascular integrity.

To conduct morphometric analysis of astrocytes and microglia, immunofluorescence (IF) staining was performed using the astrocyte marker GFAP and the microglia marker Iba1. Representative images were reconstructed in 3D. Astrocyte reactivity and proliferation were evaluated through surface analysis using Imaris 9.5.0 software (Bitplane, Switzerland). Reactive astrocytes were defined as those with a volume exceeding 200 μm^3^, while over‐activated astrocytes were characterized by a volume surpassing 600 μm^3^. Additionally, astrocytes were categorized based on the average volume: <200 μm^3^, 200–600 μm^3^, and >600 μm^3^ ^57^, ^58^ Microglia overactivation was determined by a volume exceeding 1000 μm^3^, employing surface analysis with Imaris 9.5.0 software. To obtain more detailed analysis for microglia morphology, microglial skeletonization was assessed by sholl analysis using Fiji plug‐in software in ImageJ according to previous description.^59^ Five photographs from different animals and four single microglia for each were randomly selected to be preprocessed by sharpening and noise reduction, followed by binarization. The contours of microglia cell bodies and branches were depicted with the brush tool, and the maximum and minimum radius of concentric circles were measured to analyze the number of intersections. The data from four representative intersections (the distance of 15, 20, 25, and 30 μm from soma) were used for statistical analysis and expressed as mean ± SEM, *n* = 20 for each group.

For the analysis of microvessel‐associated protein density, a 3D quantification of the proteins of interest (ZO‐1, occludin, and GLUT1) was conducted. Surface rendering of ZO‐1, occludin, or GLUT1, in conjunction with RECA1 projection, was utilized to visualize their interactions using Bitplane Imaris software. Immunostaining and analysis were performed on at least four–seven randomly selected sections per animal, and the representative images are presented.

#### 2.2.10 Transmission electron microscopy (TEM)

Transmission electron microscopy (TEM) was performed as previously described.^60^ In brief, the frontal parietal cortex was obtained in the same manner as described for the Western blot samples. The tissue samples were cut into small tissue blocks (size 1 mm^3^) and fixed with 2.5% glutaraldehyde in 0.1 M cacodylate buffer at 4°C overnight. They were then post‐fixed with 2% osmium tetroxide (OsO_4_) for 30 min and dehydrated in a series of graded ethanol solutions. Subsequently, ethanol was substituted with propylene oxide and then embedded in Epon 812. Ultrathin sections (70 nm) were mounted on 200‐mesh copper grids. The copper grids were counterstained with 2% uranyl acetate, followed by a mixture of lead nitrate–lead acetate–lead citrate. Finally, the grids were washed with PBS and distilled water. Micrographs were captured with a Tecnai Spirit G2 Twin electron microscope (FEI Co., Eindhoven, the Netherlands) at a magnification of 30,000. To determine the number of synaptic vesicles (SVs) and the extent of the postsynaptic dense area, the active zones were defined as the portion of the presynaptic membrane directly juxtaposed to the post‐synaptic density.^61^, ^62^ Three hundred synapses from each group (*n* = 3 per group) were analyzed, following the methodology outlined in a previous report.^63^ The selection of SVs was based on the presence of an active zone from the TEM images, and they were outlined in red using Adobe Photoshop software. The number of SVs per unit area (μm^2^) was then calculated by processing and analyzing the image using ImageJ. Moreover, the length (nm) of postsynaptic density per cross‐sectional area of the active zones (μm^2^) from 50 synapses was calculated.

#### 2.2.11 Behavioral assessments

##### Forced Swimming Test (FST)

The FST was conducted to assess depressive‐like behavior as previously described.^64^ In brief, the apparatus consists of a glass cylinder (60 cm height, 220 cm diameter) and an overhead camera. Rats were allowed to swim inside the cylinder filled with 30 cm deep, 23–25°C water, and videotaped for 3 min. The duration of the immobility time was counted using the ANY‐maze video tracking system. Immobility was defined as the time when animals remained floating or motionless with only movements necessary for keeping balance in the water.

##### Elevated Plus Maze (EPM)

EPM has been described as the most utilized behavioral experiment to evaluate the anxiety response of rodents.^65^ The experimental setup consisted of a 50 ‐cm‐high device, which has two opposing open arms (50 cm × 10 cm × 0.5 cm), an open platform (10 cm × 10 cm) in the center, and two opposing closed arms (50 cm × 10 cm × 30 cm). A camera placed above the center is controlled by the video tracking software (Stoelting; WoodDale, IL, USA). At the beginning of the test, the rat was placed on the central platform, facing one of the open arms, and allowed to explore for 5 min. The duration spent in the open arms and the number of times the rat entered the open arms were recorded. The maze was thoroughly cleaned with 70% ethanol between each test.

##### Open Field Task (OFT)

The OFT was used to analyze depressive and anxious behavior.^66^ In brief, the open field experimental device consists of a square open field box of 100 × 100 × 50 cm^3^ (L × W × H), with a camera placed above the center. The arena is made of black HDPE panels that are fastened together and placed on a plastic base. The experiment was performed in a darkened room, and each rat was tracked with an overhead camera controlled by video tracking software (Stoelting; WoodDale, IL, USA) for a single session of 5 min. On the first day of the OFT, allowing rats freely explored in the arena for 5 min to adapt to the environment. At the beginning of the formal test on the second day, a rat was placed at one corner of the box, making it face the wall. During a 5‐min period, the travel distance, the times of grooming and rearing, as well as time spent in the depicted central area (50 × 50 cm^2^) were recorded. Between each trial, the open‐field chamber was thoroughly cleaned with 70% ethanol to remove any odors that may affect subsequent trial performance.

##### Novel Object Recognition Test (NOR)

The novel object recognition test was conducted as previously described.^67^ In brief, the test includes two sessions: one training session and one testing session. On the first day (training session), two identical objects were placed at an equal distance in the recognition box. The rats were placed in the center of the box and allowed to explore for 5 min. On the second day (testing session), one of the familiar objects was replaced with a novel object that had the same height and volume but with a different shape and appearance. The rats were returned to the box and allowed to explore for 5 min. The ANY‐maze video‐tracking software recorded the time that the rats spent exploring each object. The discrimination index was calculated as (time devoted to novel object – time devoted to the familiar object)/(time devoted to novel object + time devoted to the familiar object) and analyzed.

##### Barnes Maze Test

As described previously,^64^ the Barnes maze test was utilized to evaluate reference memory. In brief, the Barnes maze consists of a large circular platform containing 18 holes on the outer edge and elevated approximately 120 cm above the floor. A black escape box (20 × 15 × 12 cm) is hidden under one of these holes in which the animal can hide, while the other holes are left open to the floor. During the test, an overhead light (500 W, 1000 lux) is shining down on the platform surface, and a loud (60 dB) repetitive tone is playing during the test. The Barnes maze task included three once‐daily 3‐min training trials over 3 days, followed by a 90‐s probe trial on the fourth day. During the training trail, the escape hole location was kept constant throughout the task. The rats were given a maximum 3 min to locate and enter the black escape box. The duration taken by the rat to find and enter the escape box is defined as the escape latency. If the rats failed to identify the escape hole within 3 min, they were gently guided to it and remained there for 30 s to familiarize themselves with the location. On the fourth day of the probe trial, the escape box was removed, and the corresponding hole was blocked. The time spent in the target quadrant, where the target box is located, was recorded and analyzed by ANY‐maze video tracking software (Stoelting, Wood Dale, IL, USA). Both the maze platform and escape hole were thoroughly cleaned with 70% alcohol in between each trial.

#### 2.2.12 Statistical analysis

All the experiments in this study were conducted on age‐matched female WT and APP/PS1 transgenic rats. The 12‐month‐old rats were randomly assigned to independent groups for conducting biochemical/morphological experiments and behavioral tests after the rats were ovariectomized (OVX). The data were analyzed with SigmaStat 3.5 software. Differences between normally distributed values of two experimental groups were analyzed by an unpaired Student's *t*‐test. One‐way ANOVA or two‐way ANOVA tests were conducted when there was one or more variable factors in the analysis for three groups. When the ANOVA test was found to be significant, the post‐hoc Student–Newman–Keuls (S‐N‐K) *q* test, Tukey's *t* test, or Dunnett's t test was used to make pairwise comparisons to determine the significance between multiple groups. Univariate statistical analyses were performed using the Kruskal–Wallis t‐test. Data were expressed as mean ± SEM.

## 3 RESULTS

### 3.1 Confirmation of amyloid pathology in *APP/* *PS1*‐Tg rats

The amyloid pathology of *APP/PS1*‐Tg rats was confirmed through two independent strategies. RT‐PCR results demonstrated strong expression of *APPsw* and *PS1* at 400–500 bp and 200–300 bp, respectively, in the frontoparietal cortical RNA samples of *APP/PS1*‐Tg rats (Figure S1A). Furthermore, ELISA analysis indicated a significant increase in Aβ1‐42 levels in the frontal–parietal cortex of *APP/PS1* transgenic rats as compared to WT animals, either at 3–5 months (Figure S1B; *p* = 0.0065, unpaired, 2‐tailed Student's *t*‐test) or 14–19 months (Figure S1C; *p* = 0.0045, unpaired, 2‐tailed Student's *t*‐test).

### 3.2 Remote ischemia conditioning (RIC) improved the cognitive function and alleviated depression in APP/PS1 rats

**FIGURE 2:**
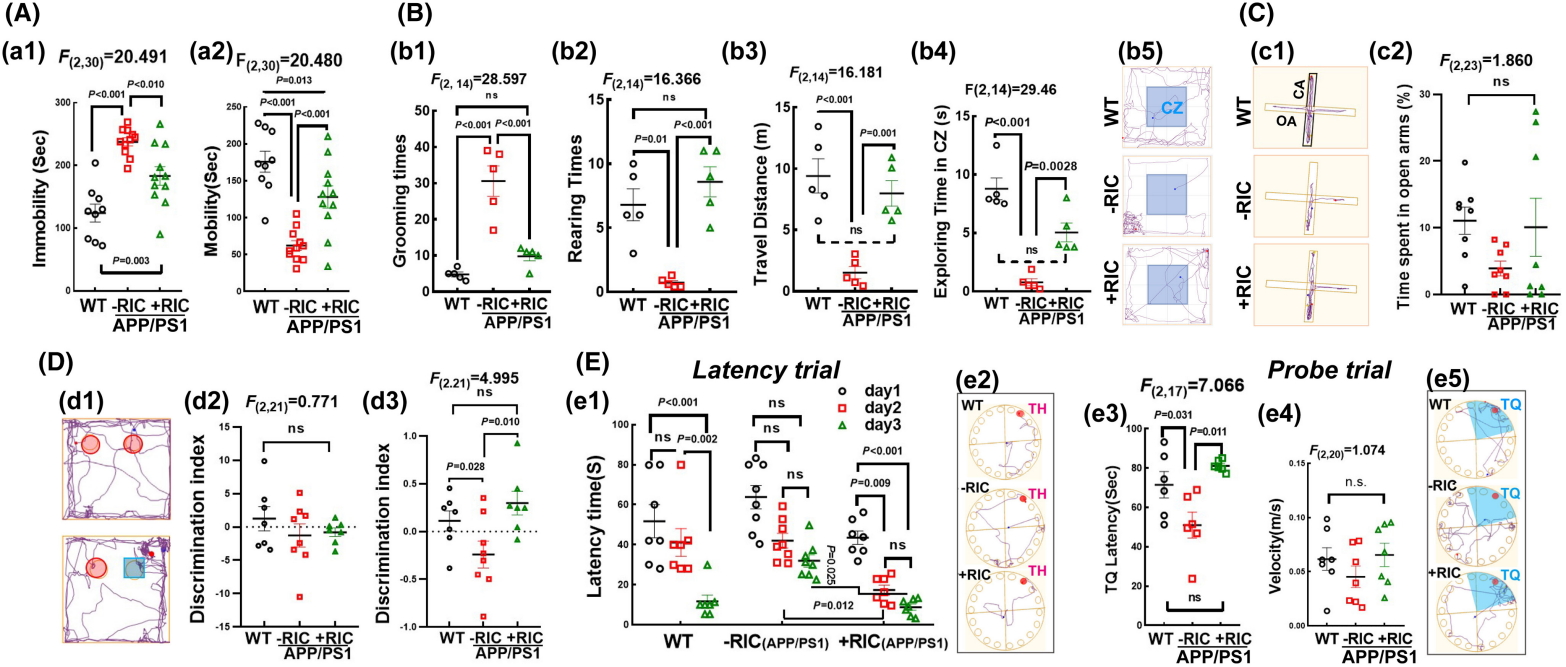
Remote ischemia conditioning promoted behavioral outcomes in OVX APP/PS1 rats. (A) Effect of RIC on the duration of immobility (a1) and mobility (swimming and climbing) (a2) behavior in the forced swim test (FST) assessed during 5 min. (B, b1–b4) Open field test (OFT) showed grooming times, rearing times, total travel distance, and exploring time in the central zone. Representative track plots of each group of rats are shown in (b5). (C) The elevated plus maze was used to detect anxiety behavior in rodents. Representative track plots are shown in (c1). Time spent in open arms of each group are shown in (c2). (D) The novel object recognition experiment evaluated the memory ability of the tested animal based on the time it spent exploring familiar and new objects. Typical track plots of each group are shown in (d1). Discrimination index can be shown in (d2, d3). (E) Barnes maze task was performed to evaluate spatial working and reference memory, which showed latency time in the training trial (e1), target quadrant latency (e3), and velocity (e4) in the probe trial. Representative track plots for the latency trial (e2) and probe trial (e5). Exploring velocity in different groups had no significant change among the three groups. Values are means ± SEM of determinations from each group. *n* = 9–11 for A; *n* = 5 for B; *n* = 6–9 for C, D and E. *n*.s., no significance. CZ, central zone; CA, close arm; OA, open arm; TH, target hole; TQ, target quadrant.

We then proceeded to assess anxiety and depressive‐like behaviors using FST, OFT, and EPM. The FST result showed that APP/PS1 rats exhibited increased total immobility time (*F*_(2,30)_ = 20.491, *p* < 0.001, 1‐way ANOVA and S‐N‐K posttest) and less mobility time (*F*_(2,30)_ = 20.491, *p* < 0.001, 1‐way ANOVA and S‐N‐K posttest) compared to WT rats. However, these changes were reversed after RIC intervention (Figure 2A, a1‐a2). Based on the OFT results, it was observed that APP/PS1 rats had considerably reduced their horizontal movement distance and mostly avoided one corner of the open field. APP/PS1 rats also spent less time standing (rearing) and more time washing their faces (grooming) as compared to WT rats (Figure 2B, b1‐b2, grooming time: *F*_(2,14)_ = 28.597, *p* < 0.001, 1‐way ANOVA and S‐N‐K posttest; rearing time: *F*_(2,14)_ = 16.366, *p* < 0.001, 1‐way ANOVA and Dunnett's posttest). The travel distance and the time exploring in the central zone (CZ) of APP/PS1 were significantly decreased compared to the WT group (Figure 2B, b3‐b4; travel distance: *F*_(2,14)_ = 16.181, *p* < 0.001, 1‐way ANOVA and S‐N‐K posttest; CZ time: *F*_(2,14)_ = 29.460, *p* < 0.001, 1‐way ANOVA and Dunnett's posttest). Notably, RIC intervention reversed these changes, showing less grooming time, as well as much longer rearing time, and total or CZ travel distance than APP/PS1 rats (Figure 2B, b1–b4). Figure 2B‐b5 shows the trace images in the OFT. The EPM test, however, shows no significant difference in the exploring time (data not shown) and the time duration spent in the OA among the three groups (Figure 2C‐c2, *F*_(2,23)_ = 1.860, *p* > 0.05, 1‐way ANOVA). Figure 2C, c1 shows the trace images in the EPM test. Taken together, these findings suggest that APP/PS1 rats exhibit depressive‐like behavior, which was significantly improved after RIC intervention.

Next, we investigated the effect of RIC on cognitive function in OVX APP/PS1 rats by performing behavioral tests. The NOR test is a relatively fast and efficient means for assessing hippocampal‐dependent recognition memory. Figure 2D, d2 shows all animals of the three groups displayed a normal object capacity (*F*_(2,21)_ = 0.771, *p* = 0.476, 1‐way ANOVA). However, APP/PS1 rats displayed a significant decrease in the novel object discrimination capacity, compared to WT animals. RIC intervention significantly reversed the effect, exhibiting a greater increase in novel object discrimination capacity (Figure 2D, d3, *F*_(2,21)_ = 4.995, *p* = 0.012, 1‐way ANOVA and S‐N‐K posttest). Figure 2D, d1 displays the representative trace images of the rats during the NOR test. Furthermore, we conducted the Barnes maze test to evaluate hippocampal‐dependent spatial reference memory. Figure 2E, e1 shows that throughout the training period (day 1–day 3), APP/PS1 rats exhibited a significant difference in the ability for locating the target hole (TH). However, WT and RIC rats spent less time finding the TH on the third day, compared to the first day of training (*p* < 0.001; two‐way ANOVA and Tukey's posttest). Compared to APP/PS1 group, RIC intervention rats spent less latency time to locate the TH on the third day of the training test (*p* = 0.0247). The statistical analysis of the probe trial revealed that the exploration time of WT rats in the quadrant where the TH was located was significantly increased compared to that of APP/PS1, and this effect was even more pronounced in RIC‐intervention animals (Figure 2E, e3, *F*_(2,17)_ = 7.066, *p* = 0.012, 1‐way ANOVA and Tukey's posttest). Figure 2E‐e2, e5 presents the trace images during the training and probe trial, respectively. The velocity of each group remained largely unchanged (Figure 2E, e4, *F*_(2,20)_ = 1.074, *p* = 0.363, 1‐way ANOVA). These findings suggest that mid‐age APP/PS1 rats displayed depressive‐like behavior and impaired learning and memory, which were significantly improved by RIC intervention.

### 3.3 RIC intervention protects cortical neurons in OVX APP/PS1 rats

**FIGURE 3:**
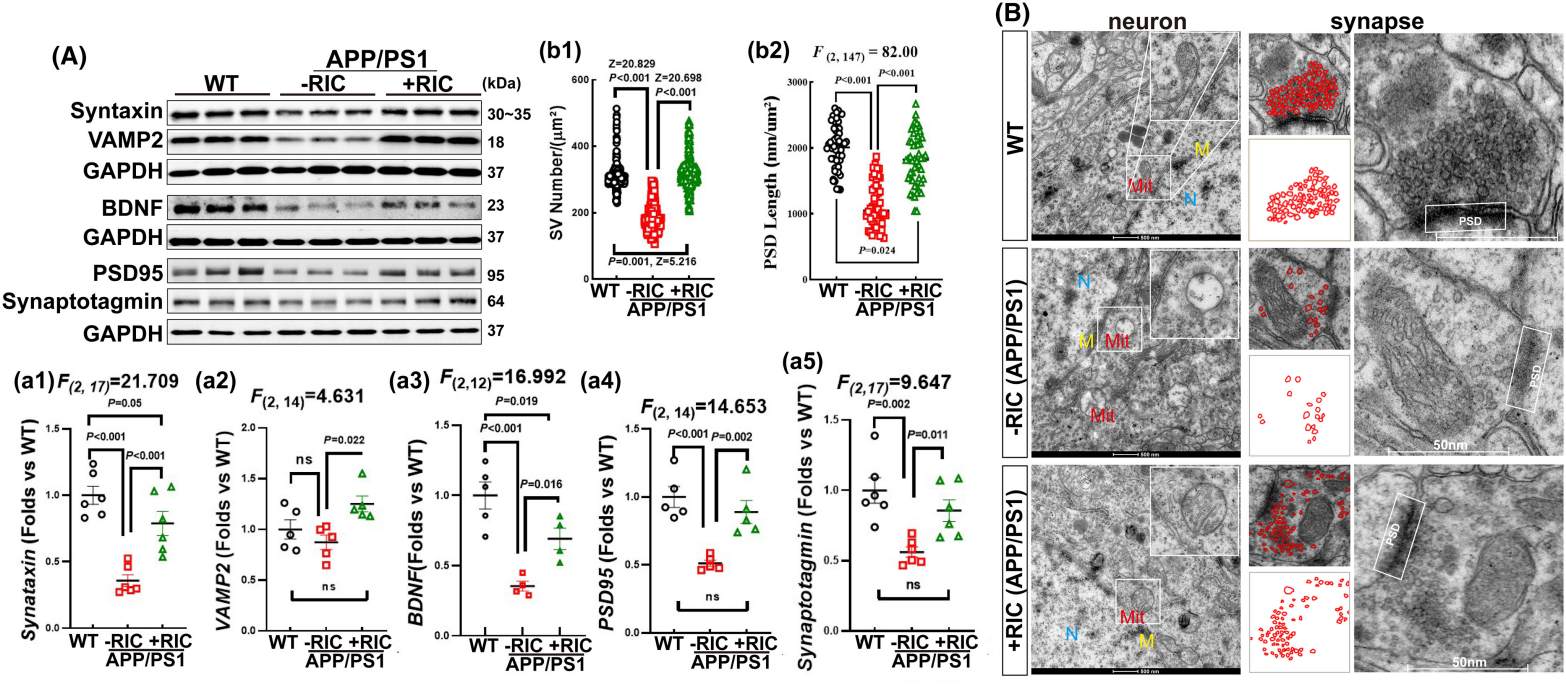
Remote ischemia conditioning protected neurons in OVX APP/PS1 rats. (A, a1–a5) Western blot analysis showed changes of synaptic‐related proteins (synaptotagmin, syntaxin, VAMP2, and PSD95), as well as cognitive‐related protein BDNF. Values are means ± SEM of determinations from each group. (B) Representative photographs of TEM of neurons and synapses in the cortex from each group. N: nuclei; Mit: mitochondria; M: nuclei membrane. The white square is an enlarged area, and the white rectangle stands for the active area of the synapse. The second column is the outline of synaptic vesicles. The changes in the number of synaptic vesicles (b1) and the length of postsynaptic dense protein (b2) are shown. All data were expressed as means ± SEM. *n* = 300 for (b1); *n* = 50 for (b2). SV, synaptic vesicle.

Neuron impairment such as synapse loss is a major contributor to cognitive dysfunction in AD. We sought to confirm the neuroprotective effects of RIC intervention by examining the neurons at the molecular level and morphology. At the molecular level, we examined the expression of various factors, including the presynaptic vesicle proteins: synaptotagmin, syntaxin, and vesicle‐associated membrane protein 1 (VAMP1), the postsynaptic marker protein PSD95, and brain‐derived neurotrophic factor (BDNF), which has been shown to play crucial roles in neuroplasticity and neuronal repair after injury. Western blot analysis showed significant decreases in the expression level of these proteins in APP/PS1 rats as compared to the WT group, while RIC intervention markedly reversed the changes (Figure 3A, a1–a5) (A‐way ANOVA and S‐N‐K posttest; syntaxin: *F*_(2,17)_ = 21.709, *p* < 0.001; VAMP2: *F*_(2,14)_ = 5.579, *p* = 0.019, *p* = 0.019; BDNF: *F*_(2,12)_ = 16.993, *p* < 0.001; PSD95: *F*_(2,14)_ = 14.653, *p* < 0.001; synaptotagmin: *F*_(2,17)_ = 9.647, *p* = 0.002). The results of TEM showed that in the cortical neurons (Figure 3B‐first column) of the APP/PS1 rats, the lipid bilayer of the nuclear membrane was compromised (N), the mitochondria ultrastructure of was vacuolated (Mit). The second and third columns demonstrated that the number of synaptic vesicles (SVs) in per μm^2^ was significantly decreased in APP/PS1 rats, compared to WT and RIC intervention groups (Figure 3B‐b1; Kruskal–Wallis, *p* < 0.001, WT vs. APP/PS1 Z = 20.829; AD vs. RIC Z = 20.698; WT vs. RIC Z = 5.216). Moreover, the length of postsynaptic dense protein was significantly decreased in APP/PS1 rats compared to WT or RIC groups (Figure 3B‐b2; *F*_(2,147)_ = 82.00, *p* < 0.001, one‐way ANOVA and Tukey's posttest). The second column displayed the outline of SVs. In contrast, RIC rats showed normal ultrastructure of neurons similar to WT rats. This was indicated by the intact ultrastructure in the nuclear membrane lipid bilayer and mitochondria cristae, as well as the higher presynaptic vesicles and expression of the postsynaptic dense protein (Figure 3B, b1‐b2).

### 3.4 RIC protects BBB integrity in the frontal–parietal cortex of middle‐aged APP/PS1 rats

**FIGURE 4:**
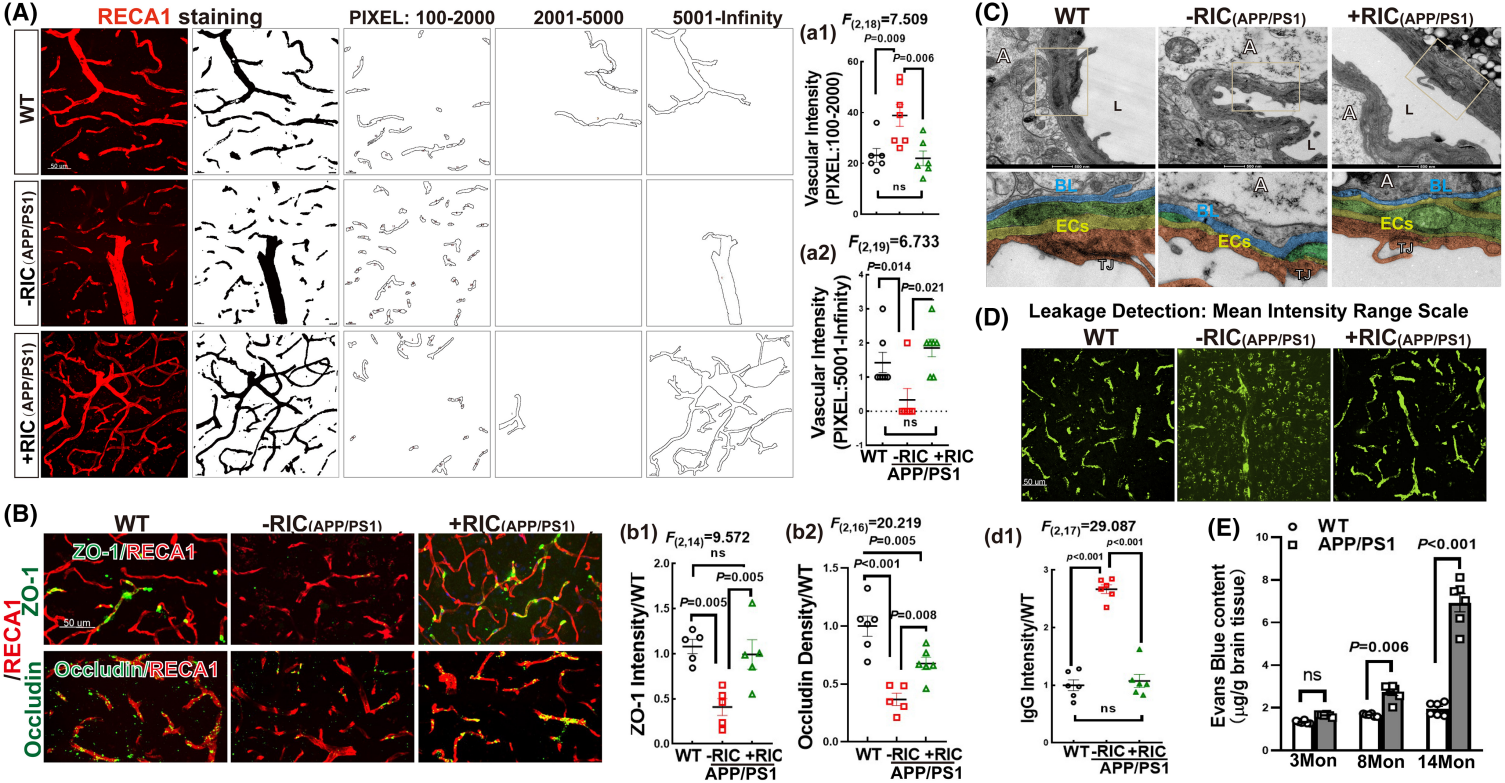
Remote ischemia conditioning protected vascular integrity in the frontal–parietal cortex of APP/PS1 OVX rats. (A, a1‐a2) Representative images showing immunofluorescence staining for RECA1 (red) and the vessel density was measured based on RECA‐1‐positive structures in the cortex. Vascular integrity was assessed according to RECA‐1‐positive structures that were thresholded into three‐pixel areas‐100–2000 (a1), 20,001–5000 (no significance between groups, statistic data was not shown), and 5001–infinity (a2) using Fiji/Image software. Data were expressed as means ± SEM. (B) Representative photos of double immunofluorescence staining for ZO‐1 with RECA1 and occludin with RECA1. Quantitative analysis of ZO‐1 (b1) and occludin (b2) fluorescence intensity expressed as fold changes vs. WT group. *n* = 5–6 for B. magnification 40 ×, scale bar 50 μm. (C) TEM results showed vascular ultrastructure in the rat cortex. Representative images of microvascular morphological changes are shown. Microvessels are compressed and narrowed by swollen astrocytic endfeet in APP/PS1 rats, while the astrocytic endfeet swelling is mild and the residual vascular lumen was well‐preserved in RIC rats. A, astrocyte; BL, basal lamina; EC, endothelial cell; L, capillary lumen; TJ, tight junction. (D) Representative images of IgG (green) among different groups. The quantitative analysis of IgG fluorescence intensity expressed as fold changes vs. WT group is shown in (d1). (E) The changes in the content of Evans blue in different groups and different ages are shown. Values are means ± SEM of determinations from each group. *N* = 6 for D; *n* = 6 for E. ns., no significance.

A recent study found that out of the top 45 genes associated with AD risk, 30 are expressed in the human brain vasculature.^68^ Actually, RIC is a kind of intermittent pressure stimulation directly on blood vessels. These observations led us to hypothesize that RIC intervention might be the preferable therapy to improve cognitive function through cerebrovascular protection. Thus, we evaluated cerebrovascular integrity using coronal sections of the rats. Immunofluorescence staining for RECA1 (red), a marker of ECs, was used to measure vessel density in the proportions of RECA1‐positive structures according to different sizes sorted into three bins by pixel area (100–2000; 2001–5000; and 5001–infinity) (Figure 4A). Quantitative analysis showed that APP/PS1 animals had more vessels with pixels 100–2000 than WT rats, while RIC intervention significantly decreased the number of small vessels (Figure 4A, a1) (*F*_(2,18)_ = 7.509, *p* = 0.005, one‐way ANOVA and S‐N‐K posttest). In addition, WT and RIC rats had much more vessels with pixels 5001–infinity than APP/PS1 rats (Figure 4A, a2) (*F*_(2,19)_ = 6.733, *p* = 0.007, a‐way ANOVA and S‐N‐K posttest). According to the findings, it was determined that the vessels in the RIC rats exhibited better Integrity than those in the APP/PS1 rats.

Moreover, double immunofluorescence staining of ZO‐1 and occludin with RECA1 in WT and RIC groups revealed that ZO‐1 and occludin expression was colocalized with RECA1 positive vessels, and their fluorescent intensity was significantly increased as compared with that of APP/PS1 rats (Figure 4B, b1‐b2) (ZO‐1: *F*_(2,14)_ = 9.572, *p* = 0.003; occludin: *F*_(2,16)_ = 20.219, *p* < 0.001; one‐way ANOVA and S‐N‐K). Furthermore, the vascular ultrastructure in the rat frontal parietal cortex was detected by TEM. The second row is the local enlarged images of the first row. As shown in Figure 4C, in APP/PS1 rats, swelling astrocytic end‐feet cover almost the entire surface of capillaries (A). Additionally, in the APP/PS1 group, the ultrastructure of basal limina (BL, blue) and endothelial cells (ECs, brown/yellow/green) was thinner, and TJ (black) was lost. The residual lumen (L) of vessels was narrowed with compression by the extensive swollen end‐feet of perivascular astrocytes. Importantly, these damages were significantly attenuated in RIC rats, exhibiting milder end‐feet swelling of astrocytes and a thicker layer of BL and PCs.

To further confirm that RIC intervention attenuated BBB permeability in OVX APP/PS1 rats, immunofluorescence staining of Ig G was performed. The results showed that Ig G‐positive staining was mostly in the vascular cells of WT and RIC rats. While, the IgG‐stained area was dramatically increased in APP/PS1 animals (Figure 4D‐d1; *F*_(2,17)_ = 29.087, *p* < 0.001; a‐way ANOVA and S‐N‐K). Finally, Evans blue leakage found that both in 8 Mon and 14 Mon APP/PS1 rats, the content of Evans blue was significantly increased compared to that of age‐matched WT rats, although it was not statistically changed in 3 Mon (Figure 4E 6 Mon: *p* = 0.006; 14 Mon: *p* < 0.001; 2‐Way ANOVA and Tukey's posttest). Taken together, the findings indicate that middle‐aged APP/PS1 rats exhibit damaged integrity and leakage of the BBB, which was mitigated by RIC intervention.

### 3.5 RIC has a positive impact on the alterations of PDGFRβ and VCAM1 in middle‐aged APP/PS1 rats

**FIGURE 5:**
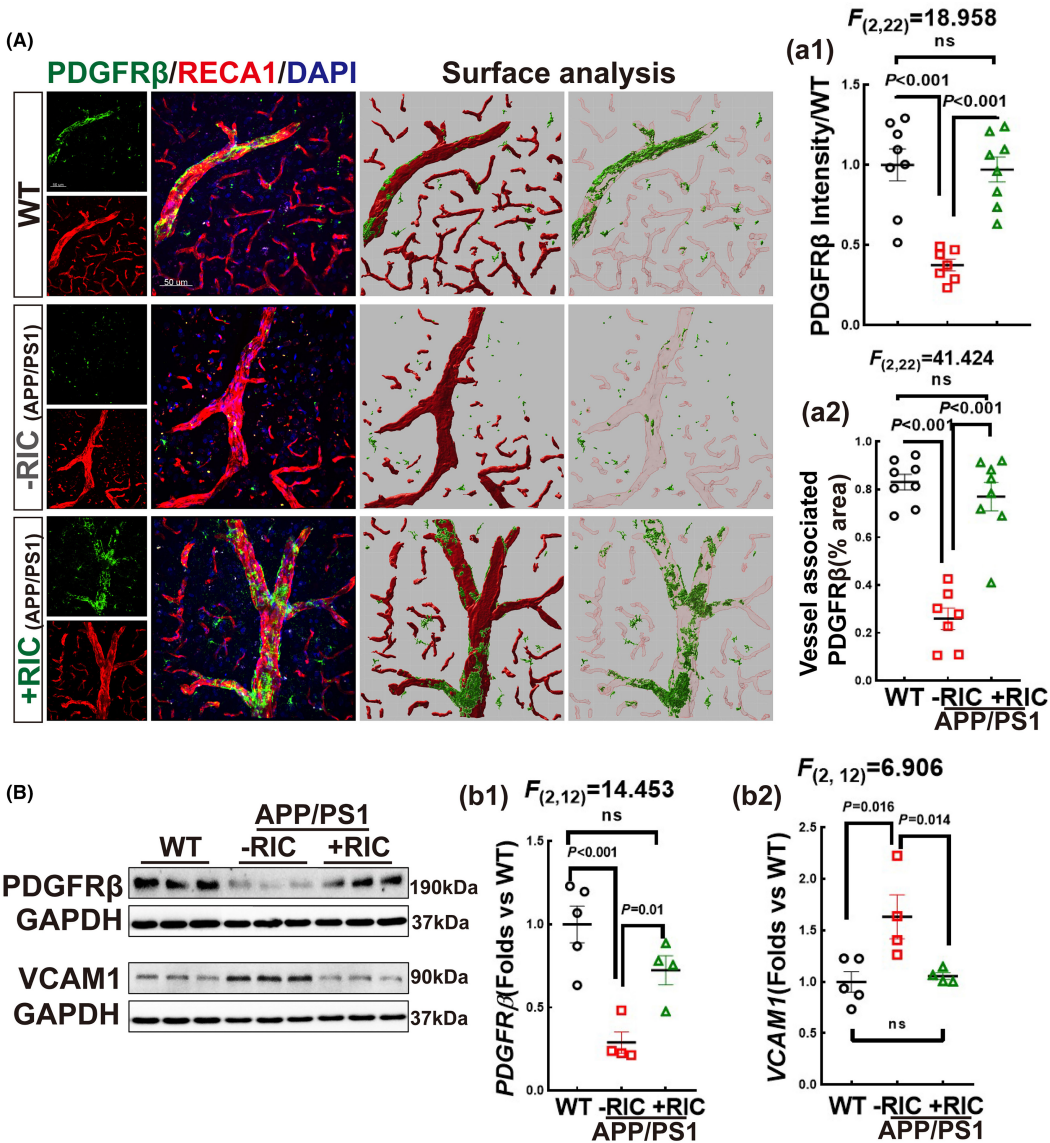
Effects of remote ischemia conditioning vascular‐related factors in the frontal–parietal cortex of OVX APP/PS1 rats. (A) Representative photographs of double immunofluorescence of PDGFRβ with RECA1. Surface analysis showed the total PDGFRβ (a1), and vessel‐associated PDGFRβ (a2), *n* = 7–8. (B, b1, b2) Western blot and data analysis for PDGFRβ and VCAM1. *N* = 4–5. Magnification 40×, scale bar 50 μm.

Pericytes are crucial components of NVU that play an essential role in maintaining the structure and function of the BBB and regulating neuroinflammation. The multi‐beneficial effects of pericytes are often mediated through ligand–receptor systems between pericytes and endothelial cells (ECs), such as PDGFβ secreted by ECs and the PDGFRβ receptor on pericytes, which is an important factor in maintaining vascular stability.^69^ Therefore, we next examined the expression of PDGFRβ using double immunofluorescence staining of PDGFRβ (green) and RECA1 (red). Surface analysis demonstrated a strong co‐localization of PDGFRβ and RECA1 (Figure 5A, a1) (*F*_(2,22)_ = 41.424, *p* < 0.001, 1‐way ANOVA and S‐N‐K). Additionally, fluorescence intensity analysis of PDGFRβ showed a significant increase in both the WT and RIC‐treated rats compared to the APP/PS1 group (Figure 5A, a2) (*F*_(2,22)_ = 18.958, *p* < 0.001, 1‐way ANOVA and S‐N‐K). Western blot analysis further confirmed that APP/PS1 rats had lower PDGFRβ protein expression than RIC and WT rats (Figure 5B, b1) (*F*_(2,12)_ = 14.453, *p* = 0.001, 1‐way ANOVA and S‐N‐K). VCAM‐1 is an essential adhesion factor in ECs and pericytes. Western blot analysis revealed that the protein expression of VCAM‐1 was significantly increased in the APP/PS1 group, as compared to WT rats, while RIC intervention significantly attenuated the increase (Figure 5B, b2) (*F*_(2,12)_ = 6.906, *p* = 0.013, 1‐way ANOVA and S‐N‐K). Together, our results indicate that RIC intervention can rescue BBB integrity in APP/PS1 rats.

### 3.6 RIC suppresses astrocyte activation and BBB leakage in the frontal–parietal cortex of APP/PS1 rats

**FIGURE 6:**
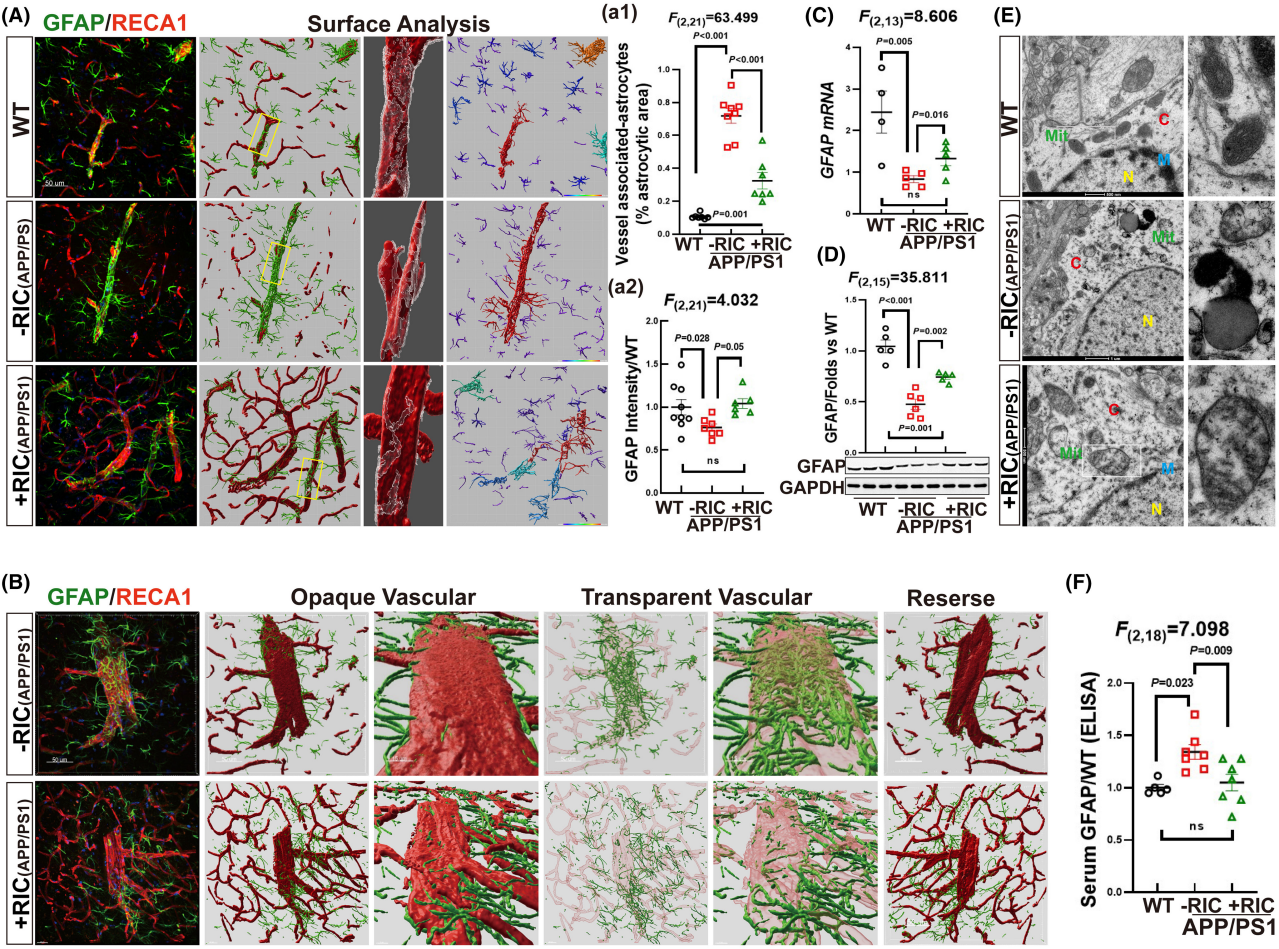
Effects of remote ischemia conditioning on astrocyte morphology and the interaction with vascular cells in the frontal–parietal cortex of OVX APP/PPS1 rats. (A) Representative photographs of double immunofluorescence staining of GFAP (green) with RECA1 (red) and the surface analysis aided by Imaris 9.5 software. Quantitative analyses of vascular‐associated GFAP in (a1) and total GFAP intensity is shown and (a2), respectively. (B) Hyalinization analysis (Opaque and Transparent) on vascular cells showed that astrocytes were mainly distributed inside the vascular lumen in the APP/PS1 rats. (C) GFAP mRNA expression by RT‐qPCR. (D) GFAP protein expression by Western blot analysis. (E) Representative photographs of astrocytes by TEM. Enlarged mitochondria from each group are shown in the second column. C, cytoplasm; M, nucleus membrane; Mit, mitochondria; N, nucleus. (F) GFAP was detected in serum by EILSA. Magnification 40×, scale bar 50 μm.

We next examined the effects of RIC on astrocytes to confirm the multi‐protective roles of RIC on NVU. We conducted double immunofluorescence staining of RECA1 (red) with GFAP (green) to observe the changes. The results showed that the fluorescent intensity of GFAP markedly declined in APP/PS1 rats as compared to the WT group, while RIC was able to rescue the decrease (Figure 6A, a1) (*F*_(2,21)_ = 4.032, *p* = 0.035, 1‐way ANOVA and S‐N‐K). We further noted that most blood vessels were tightly wrapped and compressed by astrocyte end‐feet in the cortex of APP/PS1 rats. According to the surface analysis by Imaris software, the interacting area of GFAP with RECA was found to be significantly higher in APP/PS1 rats than WT or RIC rats (Figure 6A, a2) (*F*_(2,21)_ = 63.449, *p* < 0.001, 1‐way ANOVA and S‐N‐K). Hyalinization analysis (opaque and transparent) on the blood vessels showed that in the APP/PS1 group, astrocytes were predominantly distributed inside the blood vessels. This suggests that the tightly wrapped astrocyte end‐feet may have caused vascular damage and allowed astrocytes to enter into the bloodstream (Figure 6B). To further investigate, we examined GFAP levels in circulating serum and the frontal parietal cortex. The results showed that both mRNA and protein expression of GFAP in the frontal–parietal cortex APP/PS1 rats were significantly decreased as compared to WT rats. However, RIC intervention led to a reversal of these effects, as shown in Figure 6C (*F*_(2,13)_ = 8.606, *p* = 0.006, 1‐way ANOVA and S‐N‐K) and Figure 6D (*F*_(2,15)_ = 35.811, *p* < 0.001, 1‐way ANOVA and S‐N‐K). Intriguingly, we found the GFAP level in the serum of APP rats was significantly elevated as compared to WT and RIC groups (Figure 6F *F*_(2,18)_ = 7.098, *p* = 0.006, 1‐way ANOVA and S‐N‐K). Finally, we observed the ultrastructure of astrocytes by TEM. The results showed that astrocytes of APP/PS1 rats exhibited significant pathological changes compared to the astrocytes with normal ultrastructure in WT rats. These changes included the degraded cytoplasm (C), vacuolized mitochondria (Mit), and swollen‐end feet processes. However, the damages were markedly reduced by RIC intervention, as evidenced by the mitochondria and more organelles, as shown in Figure 6E. These findings suggest that overactivation of astrocytes in the perivascular region may contribute to BBB impairment, resulting in a deteriorated intracerebral environment (homeostasis). It is worth noting that non‐invasive RIC intervention could prevent these impairments.

### 3.7 RIC promotes the astrocyte A2 phenotype in the frontal parietal cortex of APP/PS1 rats

**FIGURE 7:**
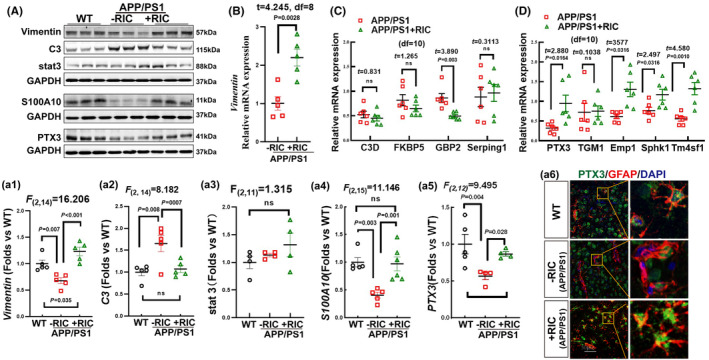
Remote ischemia conditioning reversed A1‐A2 astrocyte polarization in the frontal–parietal cortex of OVX APP/PS1 rats. (A‐a1–a5) Astrocyte activation was confirmed by examining the protein expression of PAN marker vimentin, and two typical astrocyte markers, C3 (A1), S100A10, PTX3, and Stat3 (A2) using Western blot analysis. (a6) Double immunofluorescence staining showed increased co‐localization of PTX3 with GFAP in RIC‐treated rats compared to the APP/PS1 group. (B–D) RT‐qPCR analysis showed gene levels of vimentin and A1/A2 markers of astrocytes. Values are expressed as means ± SEM of determinations from each group. *N* = 4–6, magnification 40×, scale bar 50 μm.

Recent research has classified astrocytes into two subtypes: A1 and A2. While A1 astrocytes are known to have pro‐inflammatory and neurotoxic properties, A2 astrocytes are believed to be anti‐inflammatory and neuroprotective.^70^ In view of this, we thus examined the effects of RIC on the astrocyte phenotype using antibodies to specific markers for astrocyte phenotypes (A1: C3b/c, A2: stat3, S100A10 and PTX3, PAN: vimentin). Western blot analysis showed that the protein levels of vimentin, S100A10, and PTX3 were significantly decreased in APP/PS1 rats compared to WT, while RIC markedly reversed the declines in vimentin, S100A10, and PTX3 expression (1‐way ANOVA and S‐N‐K posttest; vimentin: *F*_(2,14)_ = 16.206, *p* < 0.001; C3: *F*_(2,14)_ = 8.182, *p* = 0.006; S100A10: *F*_(2,15)_ = 11.146, *p* = 0.002; PTX3: *F*_(2,12)_ = 9.495, *p* = 0.020). Stat3 expression had no significant change among the three groups (*F*_(2,11)_ = 1.135, *p* = 0.382, 1‐way ANOVA) (Figure 7A, a1‐a5). Moreover, we selected vimentin, A1 markers (C3D, FKBP5, GBP2, Serping l), and A2 markers (PTX3, TGM1, Emp 1, Sphk 1, Tm4sf 1) to perform Q‐RT‐PCR using cortical samples. As shown in Figure 7B–D, RIC significantly decreased the mRNA level of the A1 marker GBP2 (*t* = 3.890, *p* = 0.003), while increasing the mRNA levels of the A2 markers PTX3 (*t* = 2.880, *p* = 0.0164), Emp1(*t* = 3.577, *p* = 0.0316), Sphk 1 (*t* = 2.497, *p* = 0.0316), and Tm4sf (*t* = 4.580, *p* = 0.001), as compared to the APP/PS1 group. A1 markers C3D (*t* = 0.830), FKBP5 (*t* = 1.265), and Serping 1 (*t* = 0.3113) and A2 marker TGM1 (*t* = 0.1038) had no significant change between the two groups (*p* > 0.05). The statistical significance was assessed with an unpaired two‐tailed Student's *t*‐test (GraphPad Prism). Finally, to confirm the toxic effect of the vascular‐related astrogliosis, we performed double staining for the A2 marker PTX3 (green) and GFAP (red). As shown in Figure 7A, a6, we found that PTX3 and GFAP staining was highly overlapped (yellow) in the RIC group as compared to APP/PS1 animals. These findings suggest that A2 reactive astrocytes induced by RIC might contribute to protection of BBB function in APP/PS1 rats.

### 3.8 RIC promotes GLUT1 expression in the frontal–parietal cortex of APP/PS1 rats

**FIGURE 8:**
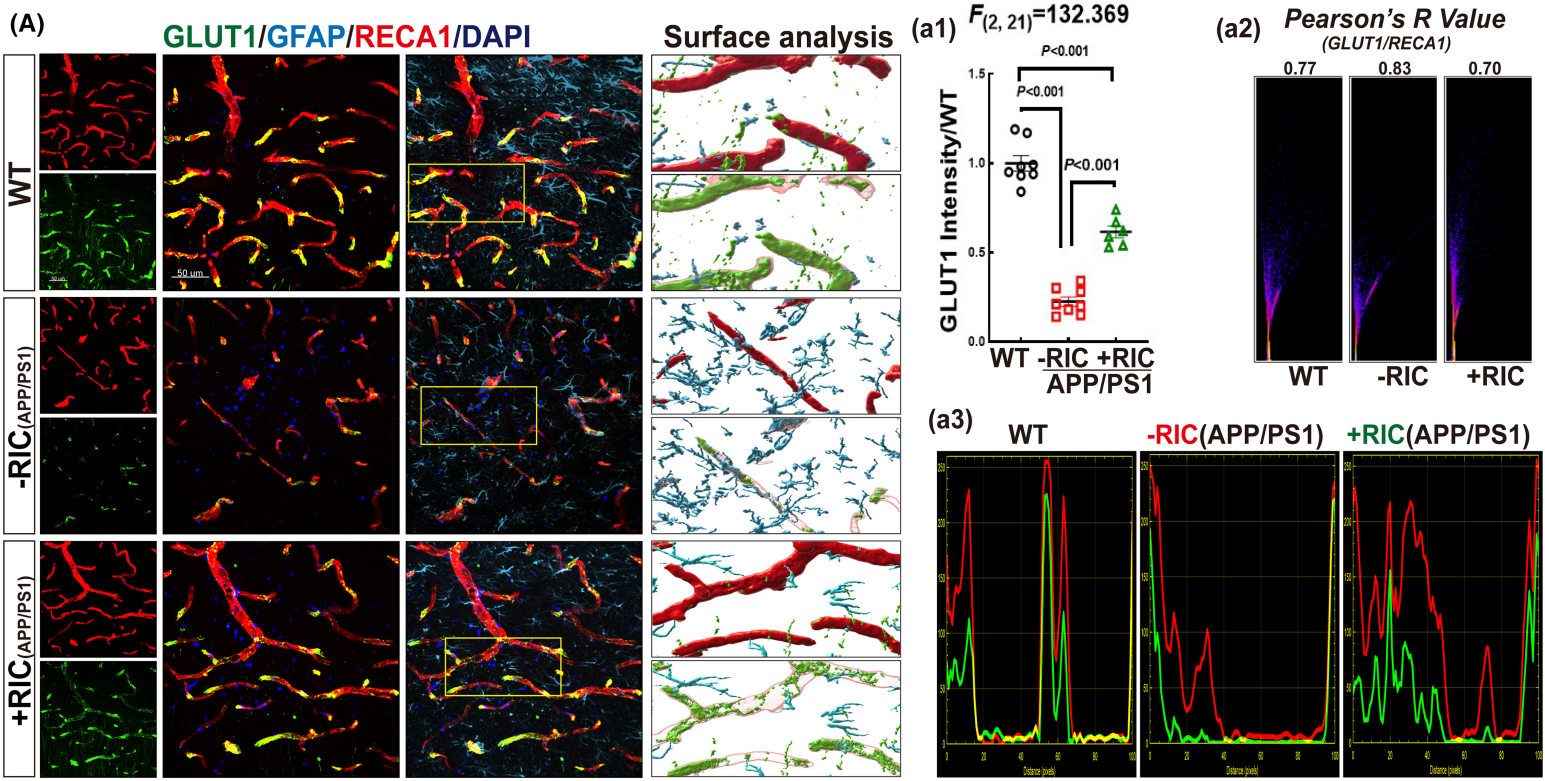
Remote ischemia conditioning upregulated GLUT1 protein expression in the frontal–parietal cortex of OVX APP/PS1 rats. (A) Representative photographs of triple immunofluorescence staining of GLUT1 (green), GFAP (blue), and RECA1 (red) and the surface analysis. (a1) Quantitative analysis of the fluorescent intensity of GLUT1+ staining. Pearson's *R*‐value analysis (a2) and colocalization (a3) between the two stained proteins for GLUT1/RECA1. *N* = 6–8, scale bar 50 μm, magnification 40×.

Perivascular foot and capillary ECs are rich in glucose and amino acid transporters to transport nutrients across the BBB and provide energy for neurons. The glucose transporter type 1 (GLUT1) is the most important energy carrier of the brain across BBB.^71^, ^72^ Endothelial GLUT1 level has been linked to microvascular impairment and BBB dysfunction in patients with Alzheimer's.^73^ Lowering GLUT1 level exacerbates AD by NVU dysfunction in mice.^74^ To further evaluate the function of the BBB, we performed triple immunofluorescence of GLUT1 (green), EC marker RECA1 (red), and astrocyte marker GFAP (blue). As shown in Figure 8A, a1, GLUT1 fluorescence intensity was significantly reduced in the cortex of APP/PS1 rats compared to the WT group, and RIC markedly upregulated GLUT1 levels (*F*_(2,11)_ = 132.369, *p* < 0.001, 1‐way ANOVA and S‐N‐K posttest). Co‐localization analysis showed a close fitting of GLUT1 and RECA1 staining, indicating the high specificity of GLUT1 expression in cerebrovascular (Figure 8A, a3). Pearson analysis was consistent with the result (Figure 8A, a2). These findings suggest that RIC intervention can prevent BBB impairment and help maintain neurovascular function.

### 3.9 RIC makes cerebrovascular integrity and increases CBF in middle‐aged APP/PS1 rats

**FIGURE 9:**
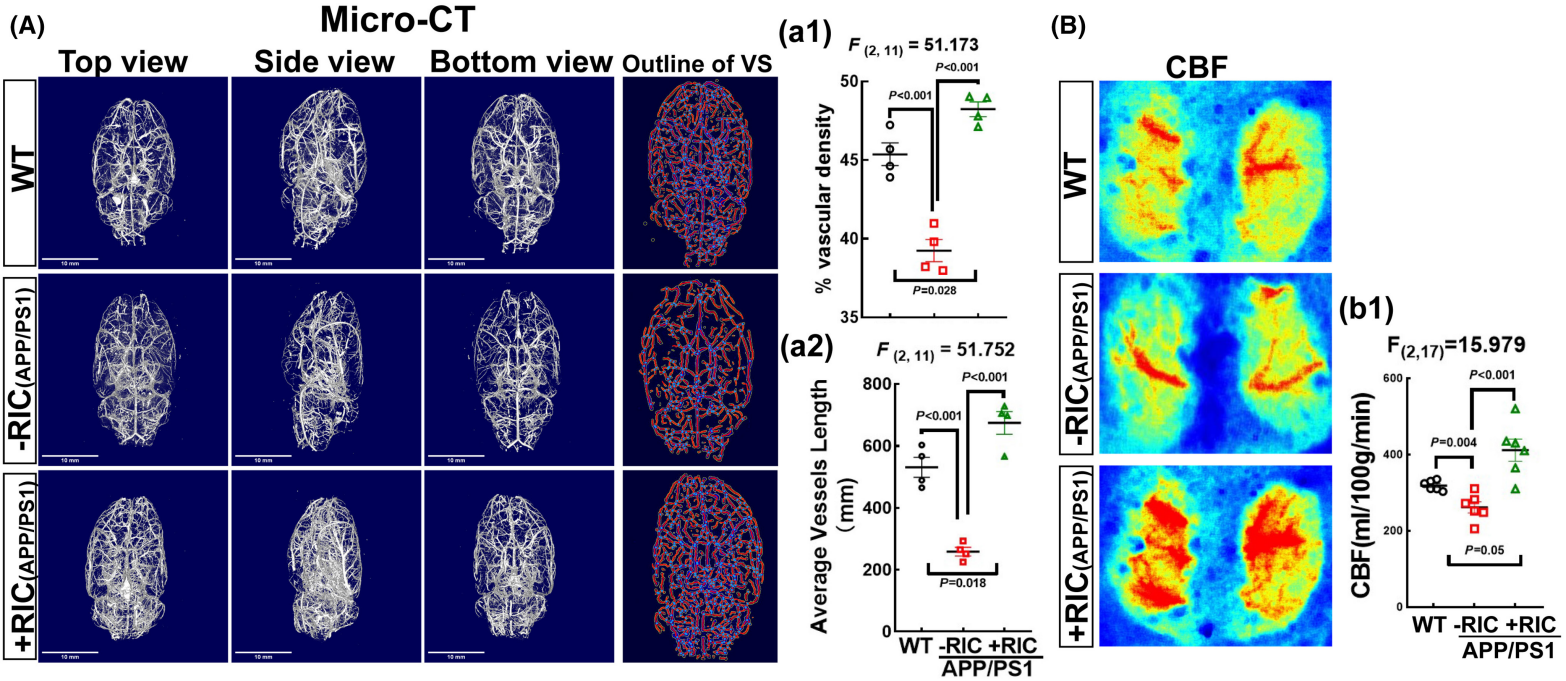
Effects of remote ischemic conditioning on cerebrovascular and cerebral blood flow in middle‐aged APP/PS1 rats. (A) Representative images of micro‐CT showed the changes in arteries, branched arteries, and capillaries in the indicated groups. The vascular density (a1) and average vessel length (a2) from four rats in each group were quantitatively analyzed. Values are expressed as means ± SEM of determinations from each group. (B, b1) CBF measurement by Speckle Laser Doppler and quantitative analysis of CBF was performed on 6–7 different animals from each group. Data were expressed as mean ± SEM.

To assess the impact of RIC intervention on cerebrovascular health, we utilized micro‐CT to observe the cerebrovascular angioarchitecture and measured CBF using Speckle Doppler. Figure 9A displays micro‐CT images in 3D visualization from different angles (top view, beside view, and bottom view) and the outline of the vascular skeleton (VS). Our quantitative analysis showed significant declines in vascular density (the percentage of vascular area divided by total brain area, Figure 9A, a1) (*F*_(2,11)_ = 51.173, *p* < 0.001, 1‐way ANOVA and Tukey posttest) and average vascular length (Figure 9A, a2) (*F*_(2,11)_ = 51.752, *p* < 0.001, 1‐way ANOVA and Tukey posttest) in APP/PS1 rats when compared to WT animals. Interestingly, RIC intervention had a profound impact on cerebrovascular health, resulting in increased vascular density and vascular length (Figure 9A‐a1, a2). Consistent with this finding, CBF in the cortex of APP/PS1 rats was significantly reduced compared to WT rats. However, RIC intervention perfectly reversed this decrease, showing a remarkable enhancement compared with APP/PS1 or WT rats (Figure 9B, b1) (*F*_(2,17)_ = 15.979, *p <* 0.001, 1‐way ANOVA and S‐N‐K posttest). Our findings confirm that RIC intervention is a promising non‐pharmaceutical intervention for AD that directly targets the cerebrovascular system, involving astrocytes, and ultimately protects cortex neurons.

### 3.10 RIC attenuates Aβ pathologies in the frontal–parietal cortex of APP/PS1 rats

**FIGURE 10:**
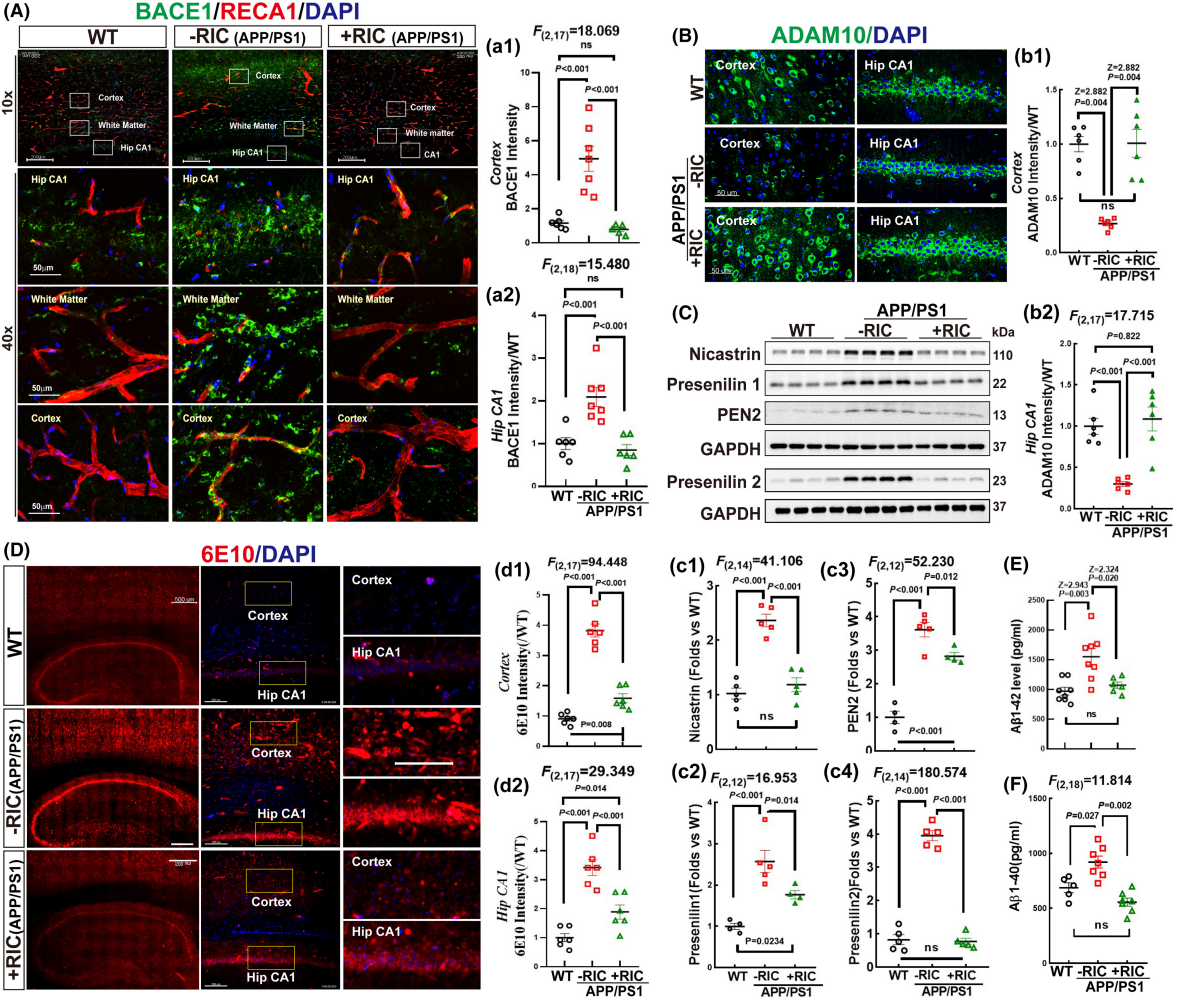
Remote ischemia conditioning suppressed Aβ production in the frontal–parietal cortex of OVX APP/PS1 rats. (A) Representative photographs of BACE1 (green) and RECA1 (red). Quantitative analysis of the fluorescence intensity of BACE1+ staining in the hippocampal CA1 (a1) and cortex (a2). Scale bar 50 μm, magnification 40×. (B) Representative photographs of ADAM10 (α‐Secretase) immunofluorescence staining (green). DAPI counterstaining of the nucleus. Quantitative analysis of the fluorescent intensity of ADAM10 in the cortex (b1), and hippocampal CA1 (b2). Scale bar 50 μm, magnification 40×. (C, c1–c4) Western blot analysis showed changes of nicastrin, presenilin 1, PEN2, and presenilin 2. Values are expressed as means ± SEM of determinations from each group. The typical photographs of 6E10 immunofluorescence staining (red) are shown in (D). Quantitative analysis of the fluorescent intensity of 6E10 in the cortex (d1), and hippocampal CA1 (d2). Scale bar 200 μm, magnification 10× for the left column; and scale bar 50 μm, magnification 40× for the right column. (E, F) The level of Aβ1‐42 and Aβ1‐40 in the cortex was detected by ELISA assay. *N* = 5–8, White Box in subfigure A. Hip CA1, hippocampal CA1.

The vasculature plays a crucial role in both the production and clearance of Aβ. Given that RIC can protect the integrity and function of the vasculature, the question arises whether RIC can also reduce the production and deposition of Aβ. To investigate, we first looked at three different proteases – α‐secretase, β‐secretase, and γ‐secretase – that are involved in the processing of APP. According to Figure 10A, a1‐a2, the intensity of BACE1, a key enzyme of APP cleavage, was higher in hippocampal CA1, white matter, and cortex compared to the WT group. RIC intervention significantly prevented the changes, leading to a decreased BACE1 level along with an intact vascular system. Additionally, more BACE1 was observed to be colocalized with RECA1 (yellow) in the APP/PS1 group, particularly in the cortex of the rats (Figure 10A) (1‐way ANOVA and S‐N‐K posttest, *p <* 0.001; Hip CA1: *F*_(2,18)_ = 15.480; Cortex: *F*_(2,17)_ = 18.069).

Furthermore, our analysis using immunofluorescence staining revealed that ADAM10, the primary α‐secretase, was expressed in both the cell membrane and cytoplasm. Interestingly, there was a notable decrease in fluorescence density in the APP/PS1 group compared to the WT rats. However, this decrease was remarkably reversed by RIC intervention (Figure 10B, b1‐b2) (Hip CA1: *F*_(2,17)_ = 17.715, 1‐way ANOVA and S‐N‐K posttest, *p <* 0.001; Cortex: WT vs. APP/PS1, Z = 2.882, *p* = 0.004; APP/PS1 vs. RIC, Z = 2.882, *p* = 0.004; WT vs. RIC, Z = 0.320, *p* = 0.749, Kruskal–Wallis t test). We also used Western blot analysis to investigate the protein expression of the γ‐secretase complex, which comprises four subunits: presenilin 1, presenilin 2, nicastrin, and presenilin enhancer protein 2 (PEN2). Our results showed that expression levels of all four proteins was significantly increased in the APP/PS1 group compared to the WT group. Importantly, RIC intervention markedly inhibited the enhancements in the γ‐secretase complex expression (Figure 10C, c1–c4) (1‐way ANOVA and S‐N‐K posttest, *p <* 0.001; nicastrin: *F*_(2,14)_ = 41.106; PEN2: *F*_(2,12)_ = 52.230; presenilin 1: *F*_(2,12)_ = 16.953; presenilin 2: *F*_(2,14)_ = 180.574).

Finally, immunofluorescence staining and ELISA were used to detect Aβ levels in brain tissue. The 6E10 antibody was used to probe total Aβ1‐42, and the results showed that the expression and distribution of 6E10 were similar to those of BACE1. The immunofluorescence staining revealed a strong perivascular fluorescent intensity in the hippocampal CA1 and cortex of APP/PS1 rats compared to the WT and RIC groups (Figure 10D, d1‐d2) (1‐way ANOVA and S‐N‐K posttest, *p <* 0.001; cortex: *F*_(2,17)_ = 94.448; Hip CA1: *F*_(2,17)_ = 29.349). The result of ELISA analysis of Aβ1‐42 was consistent with that of 6E10 staining, showing a significant increase in APP/PS1 rats versus WT and RIC groups (Figure 10E, Kruskal–Wallis *t* test, WT vs. APP/PS1, Z = 2.943, *p* = 0.004; APP/PS1 vs. RIC, Z = 2.324, *p* = 0.020; WT vs. RIC, Z = 1.034, *p* = 0.301). Additionally, ELISA measurement of Aβ1‐40 also revealed a significant increase in Aβ1‐40 levels in APP/PS1 rats compared to the WT and RIC animals (Figure 10F, 1‐way ANOVA and S‐N‐K posttest, *F*_(2,18)_ = 17.402, *p* < 0.001). Based on the above findings, it can be concluded that BBB disruption in APP/PS1 rats may increase Aβ production and decrease Aβ clearance, while RIC intervention can alleviate these impairments.

### 3.11 Remote ischemic conditioning (RIC) mitigates neuroinflammation in the frontal–parietal cortex of APP/PS1 rats

**FIGURE 11:**
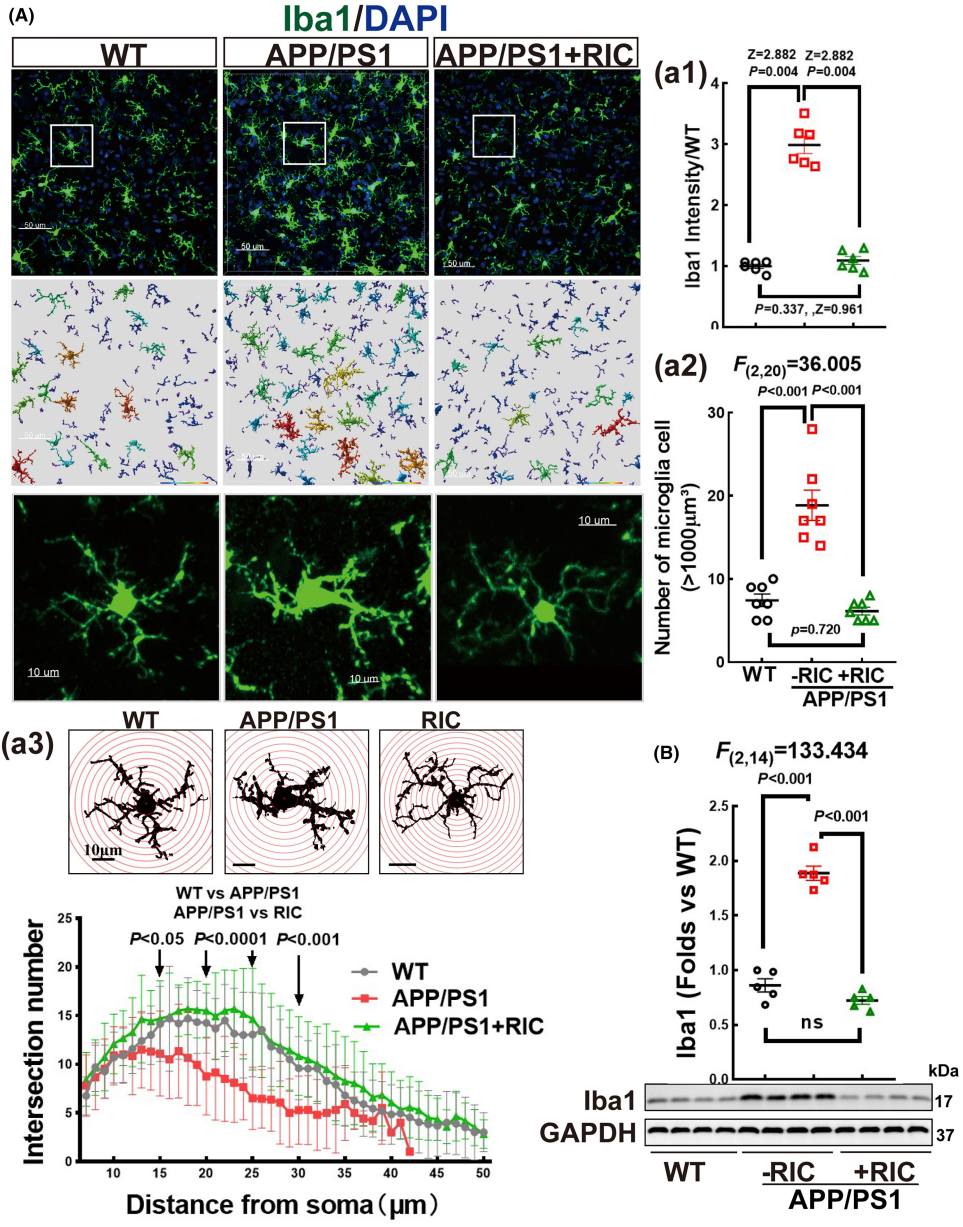
Remote ischemia conditioning suppressed microglia overactivation in the frontoparietal cortex of OVX APP/PS1 rats. (A) Representative images showed immunofluorescence staining for Iba1 (green) and surface analysis by Imaris 9.5 software. DAPI (blue) was used to count staining nuclei. Quantitative analysis of the fluorescence intensity of Iba1 (a1) and the number of microglia with a volume exceeding 1000 μm^3^ (a2). Sholl analysis of microglia profiles: inlay showed the schematic representation of the sholl analysis and main figure presented the number of intersections with the distance greater from soma in the indicated groups (a3). Values are means ± SEM of determinations from 20 single Iba1‐positive cells (*n* = 20 per groups). Magnification, 40 ×; scale bar, 50 μm for the first two rows, and 10 μm for the last row. (B) Iba1 protein expression was examined by Western blot analysis. Data were expressed as mean ± SEM, *n* = 5 in each group.

Finally, we sought to confirm whether RIC intervention could diminish microglia activation in the cortex of APP/PS1 rats. Quantification of fluorescence intensity revealed an elevated Iba1 signal (a microglia marker, depicted in green) in APP/PS1 rats compared to WT controls, and this enhancement was suppressed with RIC (Figure 11A, a1) (Z = 2.882, *p* = 0.004; Kruskal–Wallis t test). Furthermore, both surface and sholl analysis for Iba1‐positive cells indicated a significant reduction in cells exceeding 1000 μm^3^ in the RIC group compared to the APP/PS1 group (Figure 11A, a2, *p* < 0.001, *F*_(2,20)_ = 36.005, 1‐way ANOVA and S‐N‐K posttest). Additionally, microglia in the RIC intervention and WT groups exhibited more intersections at the four greater distances from the soma (Figure 11A, a3) (distance 15 μm: *F*_(2,59)_ = 4.607, WT vs. APP/PS1 *p* = 0.020, APP/PS1 vs. RIC *p* = 0.007, WT vs. RIC *p* = 0.668; distance 20 μm: *F*_(2,59)_ = 23.693, WT vs. APP/PS1 *p* < 0.0001, APP/PS1 vs. RIC *p* < 0.0001, WT vs. RIC *p* = 0.236; distance 25 μm: *F*_(2,59)_ = 19.895, WT vs. APP/PS1 *p* < 0.0001, APP/PS1 vs. RIC *p* < 0.0001, WT vs. RIC *p* = 0.247; distance 35 μm: *F*_(2,59)_ = 12.219, WT vs. APP/PS1 *p* = 0.001, APP/PS1 vs. RIC *p* < 0.001, WT vs. RIC *p* = 0.279; 1‐way ANOVA and S‐N‐K posttest). DAPI staining (depicted in blue) was employed for counting the stained nuclei. Western blot results were consistent with the fluorescence data, revealing a substantial decrease in Iba1 expression in RIC rats compared to the APP/PS1 group (Figure 11B, *p* < 0.001, *F*_(2,14)_ = 133.434, 1‐way ANOVA and S‐N‐K posttest). These findings collectively demonstrate that RIC intervention effectively alleviates inflammation in the cortex of APP/PS1 rats.

## 4 DISCUSSION

Since drug therapies can have potentially harmful side effects that can negatively affect patients, there has been an increased focus on the development of non‐pharmacological therapies for the treatment/prevention of AD. It is therefore of significant interest that our study found that RIC, a non‐pharmacological intervention, exerts multiple protective effects on the cerebrovascular, astrocytes, and neurons and improves both depressive behavior and cognitive function in middle‐aged APP/PS1 female rats. As far as we know, this is the first report on beneficial protective effects of RIC in the APP/PS1 AD transgenic rat model. In our study, we found that RIC intervention for 1 month significantly elevated CBF and profoundly protected vascular integrity. This was evidenced by the prevention of the loss of TJ proteins ZO‐1 and occludin, as well as PDGFRβ. In addition, RIC intervention also promoted capillary repair and suppression of VCAM1 levels. It should be noted that the loss of TJ proteins is a common occurrence in AD and is correlated with synaptic degeneration and AD progression.^75^ Thus, the attenuation of TJ protein loss could underlie, in part, the positive effects of RIC that were observed on the vascular system and functional outcome in our study.

Intriguingly, our study also found an altered expression of several key endothelial cell and pericyte regulatory factors such as PDGFRβ, VCAM1, and GLUT1 in APP/PS1 rats. This is important as previous work has suggested that the interplay between ECs and pericytes is crucial for maintaining the structure and function of the BBB.^76^, ^77^ Intriguingly, other studies also found alterations in these regulatory factors in AD. For example, PDGFRβ is reduced in the precuneus of AD patients,^78^ and soluble PDGFRβ levels in cerebrospinal fluid and serum have been recently recognized as a potential biomarker for early diagnosis of AD.^79^, ^80^ Furthermore, Mooradian et al also demonstrated that GLUT1 expression is reduced in AD patients as compared to age‐matched controls.^81^ Importantly, our study revealed that RIC could reverse the alterations of PDGFRβ, VCAM1, and GLUT1 expression in APP/PS1 rats. Modulation of these endothelial cell and pericyte factors thus may contribute to the beneficial effects of RIC on BBB and CBF that we observed in our study.

In addition to the regulatory roles of endothelial cells and pericytes, astrocytes also play an important role in adjusting local CBF^82^ and maintaining neuronal activity.^83^ Moreover, astrocytes are considered to be an important player in AD pathogenesis, with emerging data suggesting that plasma GFAP levels may be a promising biomarker for early AD diagnosis.^84^, ^85^, ^86^, ^87^ Therefore, it is of considerable interest that we observed several beneficial effects of RIC on astrocytes in APP/PS1 rats. These beneficial effects of RIC include (1) suppression of astrocyte overactivation and amelioration of defects in perivascular astrocytic end‐feet wrapping of the vasculature; (2) promotion of astrocyte cell proliferation, as evidenced by an increased expression of vimentin; (3) upregulating expression of markers of the A2 “neuroprotective” astrocyte phenotype, while conversely downregulating “proinflammatory” A1 astrocyte markers; and (4) marked attenuation of serum GFAP levels. Taken together, these findings indicate that in addition to regulating endothelial cell and pericyte factors and function, RIC also exerts significant beneficial effects on astrocytes that may contribute to preserving and/or promoting vascular and neuronal functions in the APP/PS1 animal model.

It should be noted that astrocytes also play a role in reducing the production of the Aβ peptide and that astrocyte‐related markers are being increasingly used as early diagnostic tools for AD.^88^, ^89^, ^90^ Interestingly, disruption of the blood–brain barrier (BBB) is also known to accelerate AD progression by impeding the removal of Aβ from the brain.^9^, ^10^ Therefore, it is noteworthy that RIC intervention was found in our study to exert positive effects on the enzymatic pathway responsible for production of Aβ peptide in APP/PS1 rats. For instance, RIC significantly elevated the expression of ADAM10, while significantly attenuating β‐secretase (BACE1) levels. ADAM10 is the main α‐secretase that cleaves APP in the non‐amyloidogenic pathway, thus inhibiting the formation of the β‐amyloid peptide^91^ Previous studies suggest that AMDM10 exerts neuroprotective effects, and FDA‐approved drugs for AD, such as memantine, can upregulate protein expression and activity of ADAM10, leading to improved cognitive function.^92^ BACE1 is the key enzyme of amyloid‐β peptide production including Aβ1‐42 and Aβ1‐40 peptides, which are implicated in initiating toxicity in AD. Therefore, BACE1 is a prime drug target for slowing down Aβ production in early AD.^93^ We also found that RIC intervention significantly suppressed the protein expression of nicastrin, PEN2, presenilin 1, and presenilin 2. These four proteins constitute the γ‐secretase complex, leading to Aβ42 and Aβ40 production. Thus, based on these results, we propose that RIC modulation of these key Aβ production enzymes is responsible for the significant decrease in Aβ1‐42 and Aβ40 levels observed in the cortex of APP/PS1 rats that received RIC intervention. Furthermore, these positive effects of RIC on proteases may also contribute to improvement of CBF and BBB function in APP/PS1 rats, as higher BACE1 activity is closely associated with CBF insufficiency and loss of TJ proteins.^94^, ^95^, ^96^, ^97^ In addition, RIC may reduce BACE1 levels, in part, by attenuating the overactivation of astrocytes, as activated astrocytes are known to release proinflammatory cytokines that facilitate BACE1 activity.^98^, ^99^, ^100^, ^101^ The extracellular accumulation of Aβ in the brain parenchyma can also induce inflammatory response, which in turn exacerbates AD progresses.^102^ Additionally, activated neuroinflammatory microglia induces A1 subtype astrocyte.^103^ Consistent with the reports, our current study revealed that APP/PS1 rats showed activated microglia and RIC intervention could suppress the inflammatory impairment. Thus, our current results strongly suggest that RIC, as a treatment with multi‐targets, could synergistically improve CBF, BBB integrity, astrocyte function, and anti‐inflammatory effect in developing AD pathology.

Finally, it is worth noting that the RIC intervention was able to prevent the loss of synaptic markers and rescue depressive‐like behavior and memory dysfunction in APP/PS1 rats. These positive effects of RIC may be attributed to the increase in TJ proteins and remodeling of astrocytes observed after RIC intervention in APP/PS1 rats. It is important to mention that BBB disruption, which is primarily due to the reduction in TJ proteins and pericyte dysfunction, can accelerate neuroinflammation and neurovascular unit structural impairment, leading to synaptic loss and cognitive deficiency.^104^, ^105^, ^106^, ^107^ Additionally, astrocytes are crucial cells that regulate neuronal synaptic plasticity.^108^ The interplay between astrocytes and neurons thus plays a vital role in shaping brain activity by regulating metabolic cooperation.^109^, ^110^ A final intriguing question is how does RIC exert its beneficial effects – e.g., how do repeated ischemic compressions of the limb muscle exert effects on the brain? In many ways, RIC effects in our study resemble beneficial effects observed previously for exercise in AD.^111^, ^112^, ^113^ Thus, it is plausible to suggest that exercise‐mediated pathways may underlie RIC effects. Indeed, recent studies have suggested that myokines, such as irisin, that are released by muscles during exercise, as well as by remote ischemic limb preconditioning, may mediate the beneficial effects of exercise and RIC on various target organs in the body.^114^, ^115^ It will therefore be intriguing in future studies to examine the possible contribution of myokines in the beneficial effects of RIC in APP/PS1 transgenic rats.

## 5 CONCLUSIONS

This study systematically elucidated the positive effects of RIC intervention on middle‐aged OVX‐APP/PS1 rats. The findings highlight the importance of prioritizing the cerebrovascular system as a crucial component for preventing and slowing AD pathologies. Additionally, the study revealed that RIC offers advantages such as improved CBF, BBB integrity, astrocyte remodeling, alleviate microglia activation and reduced Aβ peptide, while also preserving cognition during the early stages of AD. This study marks the first instance of showcasing the beneficial effects of RIC intervention in APP/PS1 rats.

## AUTHOR CONTRIBUTIONS

Ruimin Wang, Fujia Gao, and Jing Bai designed the study and supervised the experiments. Wuxiang Sun, Jiewei Hu, and Chenxu Sun participated in establishing animal models, behavioral tests, and data analysis. Yuxuan Ma, Wuxiang Sun, Chao Xu, Haoran Ma, Tao Yuan, and Zixuan Liu performed the experiments and analyzed the data. Yuanyuan Huang, Huiyu Liu, and Xin Zhang contributed to the data analysis. Wuxiang Sun, Haoran Ma, and Yuxuan Ma wrote the first draft of the manuscript, and Ruimin Wang polished the manuscript and submitted the paper. All authors have read and agreed to the published version of the manuscript.

## FUNDING INFORMATION

This study was supported by research grants from the National Natural Science Foundation of China (81671223 to RM Wang) and a key grant funded by the Science and Technology Project of Hebei Education Department (No. ZD2021087).

## CONFLICT OF INTEREST STATEMENT

The authors have declared that no competing interest exists.

## CONSENT

The paper has not been submitted and is not under consideration at any other journal. In addition, there are no related manuscripts under consideration or in press anywhere.

## Supporting information


Figure S1.

## Data Availability

Please contact Email at ruimin-wang@163.com if necessary.

## References

[1] Petersen RC , Lopez O , Armstrong MJ , et al. Practice guideline update summary: mild cognitive impairment: report of the guideline development, dissemination, and implementation Subcommittee of the American Academy of neurology. Neurology. 2018;90:126‐135. doi:10.1212/wnl.0000000000004826 29282327 PMC5772157

[2] Ward A , Tardiff S , Dye C , Arrighi HM . Rate of conversion from prodromal Alzheimer's disease to Alzheimer's dementia: a systematic review of the literature. Dement Geriatr Cogn Dis Extra. 2013;3:320‐332. doi:10.1159/000354370 24174927 PMC3808216

[3] 2022 Alzheimer's disease facts and figures. Alzheimers Dement. 2022;18:700‐789. doi:10.1002/alz.12638 35289055

[4] Bloom GS . Amyloid‐β and tau: the trigger and bullet in Alzheimer disease pathogenesis. JAMA Neurol. 2014;71:505‐508. doi:10.1001/jamaneurol.2013.5847 24493463 PMC12908160

[5] Rabbito A , Dulewicz M , Kulczyńska‐Przybik A , Mroczko B . Biochemical markers in Alzheimer's disease. Int J Mol Sci. 2020;21:21. doi:10.3390/ijms21061989 PMC7139967 32183332

[6] Livingston G , Huntley J , Sommerlad A , et al. Dementia prevention, intervention, and care: 2020 report of the lancet commission. Lancet. 2020;396:413‐446. doi:10.1016/s0140-6736(20)30367-6 32738937 PMC7392084

[7] Gautam J , Yao Y . Roles of Pericytes in stroke pathogenesis. Cell Transplant. 2018;27:1798‐1808. doi:10.1177/0963689718768455 29845887 PMC6300777

[8] Obermeier B , Daneman R , Ransohoff RM . Development, maintenance and disruption of the blood‐brain barrier. Nat Med. 2013;19:1584‐1596. doi:10.1038/nm.3407 24309662 PMC4080800

[9] Bell RD , Zlokovic BV . Neurovascular mechanisms and blood‐brain barrier disorder in Alzheimer's disease. Acta Neuropathol. 2009;118:103‐113. doi:10.1007/s00401-009-0522-3 19319544 PMC2853006

[10] Ma Q , Zhao Z , Sagare AP , et al. Blood‐brain barrier‐associated pericytes internalize and clear aggregated amyloid‐β42 by LRP1‐dependent apolipoprotein E isoform‐specific mechanism. Mol Neurodegener. 2018;13:57. doi:10.1186/s13024-018-0286-0 30340601 PMC6194676

[11] Berthiaume AA , Schmid F , Stamenkovic S , et al. Pericyte remodeling is deficient in the aged brain and contributes to impaired capillary flow and structure. Nat Commun. 2022;13:5912. doi:10.1038/s41467-022-33464-w 36207315 PMC9547063

[12] Sagare AP , Bell RD , Zhao Z , et al. Pericyte loss influences Alzheimer‐like neurodegeneration in mice. Nat Commun. 2013;4:2932. doi:10.1038/ncomms3932 24336108 PMC3945879

[13] Halliday MR , Rege SV , Ma Q , et al. Accelerated pericyte degeneration and blood‐brain barrier breakdown in apolipoprotein E4 carriers with Alzheimer's disease. J Cereb Blood Flow Metab. 2016;36:216‐227. doi:10.1038/jcbfm.2015.44 25757756 PMC4758554

[14] Zhang YL , Wang J , Zhang ZN , Su Q , Guo JH . The relationship between amyloid‐beta and brain capillary endothelial cells in Alzheimer's disease. Neural Regen Res. 2022;17:2355‐2363. doi:10.4103/1673-5374.335829 35535871 PMC9120708

[15] Bosson A , Paumier A , Boisseau S , Jacquier‐Sarlin M , Buisson A , Albrieux M . TRPA1 channels promote astrocytic Ca(2+) hyperactivity and synaptic dysfunction mediated by oligomeric forms of amyloid‐β peptide. Mol Neurodegener. 2017;12:53. doi:10.1186/s13024-017-0194-8 28683776 PMC5501536

[16] Cummings J , Lee G , Nahed P , et al. Alzheimer's disease drug development pipeline: 2022. Alzheimers Dement (N Y). 2022;8:e12295. doi:10.1002/trc2.12295 35516416 PMC9066743

[17] Aducanumab DS . First approval. Drugs. 2021;81:1437‐1443. doi:10.1007/s40265-021-01569-z 34324167

[18] Budd Haeberlein S , Aisen PS , Barkhof F , et al. Two randomized phase 3 studies of Aducanumab in early Alzheimer's disease. J Prev Alzheimers Dis. 2022;9:197‐210. doi:10.14283/jpad.2022.30 35542991

[19] Salloway S , Chalkias S , Barkhof F , et al. Amyloid‐related imaging abnormalities in 2 phase 3 studies evaluating Aducanumab in patients with early Alzheimer disease. JAMA Neurol. 2022;79:13‐21. doi:10.1001/jamaneurol.2021.4161 34807243 PMC8609465

[20] Dirnagl U , Becker K , Meisel A . Preconditioning and tolerance against cerebral ischaemia: from experimental strategies to clinical use. Lancet Neurol. 2009;8:398‐412. doi:10.1016/s1474-4422(09)70054-7 19296922 PMC2668955

[21] McDonald MW , Dykes A , Jeffers MS , et al. Remote ischemic conditioning and stroke recovery. Neurorehabil Neural Repair. 2021;35:545‐549. doi:10.1177/15459683211011224 33955298 PMC8135236

[22] García Del Blanco B , Otaegui I , Rodríguez‐Palomares JF , et al. Effect of COMBinAtion therapy with remote ischemic conditioning and exenatide on the myocardial infarct size: a two‐by‐two factorial randomized trial (COMBAT‐MI). Basic Res Cardiol. 2021;116:4. doi:10.1007/s00395-021-00842-2 33495853

[23] Hess NC , Smart NA . Isometric exercise training for managing vascular risk factors in mild cognitive impairment and Alzheimer's disease. Front Aging Neurosci. 2017;9:48. doi:10.3389/fnagi.2017.00048 28316570 PMC5334511

[24] Lang JA , Kim J , Franke WD , Vianna LC . Seven consecutive days of remote ischaemic preconditioning improves cutaneous vasodilatory capacity in young adults. J Physiol. 2019;597:757‐765. doi:10.1113/jp277185 30506681 PMC6355625

[25] Surkar SM , Bland MD , Mattlage AE , et al. Effects of remote limb ischemic conditioning on muscle strength in healthy young adults: a randomized controlled trial. PLoS One. 2020;15:e0227263. doi:10.1371/journal.pone.0227263 32017777 PMC6999897

[26] Sutter EN , Mattlage AE , Bland MD , et al. Remote limb ischemic conditioning and motor learning: evaluation of factors influencing response in older adults. Transl Stroke Res. 2019;10:362‐371. doi:10.1007/s12975-018-0653-8 30088217 PMC6367068

[27] You J , Feng L , Bao L , Xin M , Ma D , Feng J . Potential applications of remote limb ischemic conditioning for chronic cerebral circulation insufficiency. Front Neurol. 2019;10:467. doi:10.3389/fneur.2019.00467 31130914 PMC6509171

[28] Yang J , Liu C , Du X , et al. Hypoxia inducible factor 1α plays a key role in remote ischemic preconditioning against stroke by modulating inflammatory responses in rats. J Am Heart Assoc. 2018;7:7. doi:10.1161/jaha.117.007589 PMC5866324 29478025

[29] Yang J , Balkaya M , Beltran C , Heo JH , Cho S . Remote Postischemic conditioning promotes stroke recovery by shifting circulating monocytes to CCR2(+) Proinflammatory subset. J Neurosci. 2019;39:7778‐7789. doi:10.1523/jneurosci.2699-18.2019 31427395 PMC6764204

[30] Wei M , Xin P , Li S , et al. Repeated remote ischemic postconditioning protects against adverse left ventricular remodeling and improves survival in a rat model of myocardial infarction. Circ Res. 2011;108:1220‐1225. doi:10.1161/circresaha.110.236190 21474817

[31] Liu ZJ , Chen C , Li XR , et al. Remote ischemic preconditioning‐mediated neuroprotection against stroke is associated with significant alterations in peripheral immune responses. CNS Neurosci Ther. 2016;22:43‐52. doi:10.1111/cns.12448 26384716 PMC6492849

[32] Hummitzsch L , Zitta K , Fritze L , et al. Effects of remote ischemic preconditioning (RIPC) and chronic remote ischemic preconditioning (cRIPC) on levels of plasma cytokines, cell surface characteristics of monocytes and in‐vitro angiogenesis: a pilot study. Basic Res Cardiol. 2021;116:60. doi:10.1007/s00395-021-00901-8 34651218 PMC8516789

[33] Hess DC , Blauenfeldt RA , Andersen G , et al. Remote ischaemic conditioning‐a new paradigm of self‐protection in the brain. Nat Rev Neurol. 2015;11:698‐710. doi:10.1038/nrneurol.2015.223 26585977

[34] Tom SE , Hubbard RA , Crane PK , et al. Characterization of dementia and Alzheimer's disease in an older population: updated incidence and life expectancy with and without dementia. Am J Public Health. 2015;105:408‐413. doi:10.2105/ajph.2014.301935 25033130 PMC4318311

[35] Scott EL , Zhang QG , Vadlamudi RK , Brann DW . Premature menopause and risk of neurological disease: basic mechanisms and clinical implications. Mol Cell Endocrinol. 2014;389:2‐6. doi:10.1016/j.mce.2014.01.013 24462786 PMC4040297

[36] Shuster LT , Rhodes DJ , Gostout BS , Grossardt BR , Rocca WA . Premature menopause or early menopause: long‐term health consequences. Maturitas. 2010;65:161‐166. doi:10.1016/j.maturitas.2009.08.003 19733988 PMC2815011

[37] Bove R , Secor E , Chibnik LB , et al. Age at surgical menopause influences cognitive decline and Alzheimer pathology in older women. Neurology. 2014;82:222‐229. doi:10.1212/wnl.0000000000000033 24336141 PMC3902759

[38] Yusa K , Rad R , Takeda J , Bradley A . Generation of transgene‐free induced pluripotent mouse stem cells by the piggyBac transposon. Nat Methods. 2009;6:363‐369. doi:10.1038/nmeth.1323 19337237 PMC2677165

[39] Chang H , Pan Y , Landrette S , et al. Efficient genome‐wide first‐generation phenotypic screening system in mice using the piggyBac transposon. Proc Natl Acad Sci USA. 2019;116:18507‐18516. doi:10.1073/pnas.1906354116 31451639 PMC6744845

[40] Zhao S , Jiang E , Chen S , et al. PiggyBac transposon vectors: the tools of the human gene encoding. Transl Lung Cancer Res. 2016;5:120‐125. doi:10.3978/j.issn.2218-6751.2016.01.05 26958506 PMC4758974

[41] Jin Y , Chen Y , Zhao S , et al. DNA‐PK facilitates piggyBac transposition by promoting paired‐end complex formation. Proc Natl Acad Sci USA. 2017;114:7408‐7413. doi:10.1073/pnas.1612980114 28645898 PMC5514698

[42] Elick TA , Bauser CA , Fraser MJ . Excision of the piggyBac transposable element in vitro is a precise event that is enhanced by the expression of its encoded transposase. Genetica. 1996;98:33‐41. doi:10.1007/bf00120216 8765680

[43] Sandoval‐Villegas N , Nurieva W , Amberger M , Ivics Z . Contemporary transposon tools: a review and guide through mechanisms and applications of sleeping beauty, piggyBac and Tol2 for genome engineering. Int J Mol Sci. 2021;22:22. doi:10.3390/ijms22105084 PMC8151067 34064900

[44] Gu J , Sumer H , Cromer B . Efficient generation of stable cell lines with inducible neuronal transgene expression using the piggyBac transposon system. Methods Mol Biol. 2022;2495:49‐66. doi:10.1007/978-1-0716-2301-5_3 35696027

[45] Lacoste A , Berenshteyn F , Brivanlou AH . An efficient and reversible transposable system for gene delivery and lineage‐specific differentiation in human embryonic stem cells. Cell Stem Cell. 2009;5:332‐342. doi:10.1016/j.stem.2009.07.011 19733544

[46] Du X , Yang J , Liu C , et al. Hypoxia‐inducible factor 1α and 2α have beneficial effects in remote ischemic preconditioning against stroke by modulating inflammatory responses in aged rats. Front Aging Neurosci. 2020;12:54. doi:10.3389/fnagi.2020.00054 32210788 PMC7076079

[47] Wan T , Zhu W , Zhao Y , et al. Astrocytic phagocytosis contributes to demyelination after focal cortical ischemia in mice. Nat Commun. 2022;13:1134. doi:10.1038/s41467-022-28777-9 35241660 PMC8894352

[48] Wang HK , Wang YX , Xue CB , et al. Angiogenesis in tissue‐engineered nerves evaluated objectively using MICROFIL perfusion and micro‐CT scanning. Neural Regen Res. 2016;11:168‐173. doi:10.4103/1673-5374.175065 26981108 PMC4774213

[49] Ghanavati S , Yu LX , Lerch JP , Sled JG . A perfusion procedure for imaging of the mouse cerebral vasculature by X‐ray micro‐CT. J Neurosci Methods. 2014;221:70‐77. doi:10.1016/j.jneumeth.2013.09.002 24056228

[50] Citro A , Neroni A , Pignatelli C , et al. Directed self‐assembly of a xenogeneic vascularized endocrine pancreas for type 1 diabetes. Nat Commun. 2023;14:878. doi:10.1038/s41467-023-36582-1 36797282 PMC9935529

[51] Nording H , Baron L , Haberthür D , et al. The C5a/C5a receptor 1 axis controls tissue neovascularization through CXCL4 release from platelets. Nat Commun. 2021;12:3352. doi:10.1038/s41467-021-23499-w 34099640 PMC8185003

[52] Rota A , Possenti L , Offeddu GS , et al. A three‐dimensional method for morphological analysis and flow velocity estimation in microvasculature on‐a‐chip. Bioeng Transl Med. 2023;8:e10557. doi:10.1002/btm2.10557 37693050 PMC10487341

[53] Zudaire E , Gambardella L , Kurcz C , Vermeren S . A computational tool for quantitative analysis of vascular networks. PLoS One. 2011;6:e27385. doi:10.1371/journal.pone.0027385 22110636 PMC3217985

[54] Zhang X , Fan Z , Jin T . Crocin protects against cerebral‐ ischemia‐induced damage in aged rats through maintaining the integrity of blood‐brain barrier. Restor Neurol Neurosci. 2017;35:65‐75. doi:10.3233/rnn-160696 28059805

[55] Bai N , Zhang Q , Zhang W , et al. G‐protein‐coupled estrogen receptor activation upregulates interleukin‐1 receptor antagonist in the hippocampus after global cerebral ischemia: implications for neuronal self‐defense. J Neuroinflammation. 2020;17:45. doi:10.1186/s12974-020-1715-x 32007102 PMC6995076

[56] Cavaglia M , Dombrowski SM , Drazba J , Vasanji A , Bokesch PM , Janigro D . Regional variation in brain capillary density and vascular response to ischemia. Brain Res. 2001;910:81‐93. doi:10.1016/s0006-8993(01)02637-3 11489257

[57] Lu Y , Sareddy GR , Wang J , et al. Neuron‐derived estrogen is critical for astrocyte activation and neuroprotection of the ischemic brain. J Neurosci. 2020;40:7355‐7374. doi:10.1523/jneurosci.0115-20.2020 32817249 PMC7534920

[58] Wang L , Liu J , Xu J , Zhang W , Wang R . Coupling of GPR30 mediated neurogenesis and protection with astroglial aromatase‐STAT3 signaling in rat hippocampus after global cerebral ischemia. Mol Cell Endocrinol. 2021;535:111394. doi:10.1016/j.mce.2021.111394 34274445

[59] Binley KE , Ng WS , Tribble JR , Song B , Morgan JE . Sholl analysis: a quantitative comparison of semi‐automated methods. J Neurosci Methods. 2014;225:65‐70. doi:10.1016/j.jneumeth.2014.01.017 24485871

[60] Zhou C , Tu J , Zhang Q , et al. Delayed ischemic postconditioning protects hippocampal CA1 neurons by preserving mitochondrial integrity via Akt/GSK3β signaling. Neurochem Int. 2011;59:749‐758. doi:10.1016/j.neuint.2011.08.008 21867737

[61] Toni N , Buchs PA , Nikonenko I , Povilaitite P , Parisi L , Muller D . Remodeling of synaptic membranes after induction of long‐term potentiation. J Neurosci. 2001;21:6245‐6251. doi:10.1523/jneurosci.21-16-06245.2001 11487647 PMC6763190

[62] Patzke C , Dai J , Brockmann MM , et al. Cannabinoid receptor activation acutely increases synaptic vesicle numbers by activating synapsins in human synapses. Mol Psychiatry. 2021;26:6253‐6268. doi:10.1038/s41380-021-01095-0 33931733

[63] Sorrells SF , Paredes MF , Cebrian‐Silla A , et al. Human hippocampal neurogenesis drops sharply in children to undetectable levels in adults. Nature. 2018;555:377‐381. doi:10.1038/nature25975 29513649 PMC6179355

[64] Wu C , Yang L , Li Y , et al. Effects of exercise training on anxious‐depressive‐like behavior in Alzheimer rat. Med Sci Sports Exerc. 2020;52:1456‐1469. doi:10.1249/mss.0000000000002294 32028456 PMC8015320

[65] Walf AA , Frye CA . The use of the elevated plus maze as an assay of anxiety‐related behavior in rodents. Nat Protoc. 2007;2:322‐328. doi:10.1038/nprot.2007.44 17406592 PMC3623971

[66] Wong RS , Cechetto DF , Whitehead SN . Assessing the effects of acute amyloid beta oligomer exposure in the rat. Int J Mol Sci. 2016;17:17. doi:10.3390/ijms17091390 PMC5037670 27563885

[67] Lueptow LM . Novel object recognition test for the investigation of learning and memory in mice. J Vis Exp. 2017;126. doi:10.3791/55718 PMC5614391 28892027

[68] Yang AC , Vest RT , Kern F , et al. A human brain vascular atlas reveals diverse mediators of Alzheimer's risk. Nature. 2022;603:885‐892. doi:10.1038/s41586-021-04369-3 35165441 PMC9635042

[69] Bhowmick S , D'Mello V , Caruso D , Wallerstein A , Abdul‐Muneer PM . Impairment of pericyte‐endothelium crosstalk leads to blood‐brain barrier dysfunction following traumatic brain injury. Exp Neurol. 2019;317:260‐270. doi:10.1016/j.expneurol.2019.03.014 30926390

[70] Ding ZB , Song LJ , Wang Q , Kumar G , Yan YQ , Ma CG . Astrocytes: a double‐edged sword in neurodegenerative diseases. Neural Regen Res. 2021;16:1702‐1710. doi:10.4103/1673-5374.306064 33510058 PMC8328766

[71] Kolic I , Radic Nisevic J , Vlasic Cicvaric I , et al. GLUT1 deficiency syndrome‐early treatment maintains cognitive development? Genes (Basel). 2021;12:12. doi:10.3390/genes12091379 PMC8472230 34573360

[72] Koch H , Weber YG . The glucose transporter type 1 (Glut1) syndromes. Epilepsy Behav. 2019;91:90‐93. doi:10.1016/j.yebeh.2018.06.010 30076047

[73] Kalaria RN , Harik SI . Reduced glucose transporter at the blood‐brain barrier and in cerebral cortex in Alzheimer disease. J Neurochem. 1989;53:1083‐1088. doi:10.1111/j.1471-4159.1989.tb07399.x 2769254

[74] Winkler EA , Nishida Y , Sagare AP , et al. GLUT1 reductions exacerbate Alzheimer's disease vasculo‐neuronal dysfunction and degeneration. Nat Neurosci. 2015;18:521‐530. doi:10.1038/nn.3966 25730668 PMC4734893

[75] Yamazaki Y , Shinohara M , Shinohara M , et al. Selective loss of cortical endothelial tight junction proteins during Alzheimer's disease progression. Brain. 2019;142:1077‐1092. doi:10.1093/brain/awz011 30770921 PMC6439325

[76] Chiaverina G , di Blasio L , Monica V , et al. Dynamic interplay between Pericytes and endothelial cells during sprouting angiogenesis. Cell. 2019;8:8. doi:10.3390/cells8091109 PMC6770602 31546913

[77] Procter TV , Williams A , Montagne A . Interplay between brain Pericytes and endothelial cells in dementia. Am J Pathol. 2021;191:1917‐1931. doi:10.1016/j.ajpath.2021.07.003 34329605

[78] Miners JS , Schulz I , Love S . Differing associations between Aβ accumulation, hypoperfusion, blood‐brain barrier dysfunction and loss of PDGFRB pericyte marker in the precuneus and parietal white matter in Alzheimer's disease. J Cereb Blood Flow Metab. 2018;38:103‐115. doi:10.1177/0271678x17690761 28151041 PMC5757436

[79] De Kort AM , Kuiperij HB , Kersten I , et al. Normal cerebrospinal fluid concentrations of PDGFRβ in patients with cerebral amyloid angiopathy and Alzheimer's disease. Alzheimers Dement. 2022;18:1788‐1796. doi:10.1002/alz.12506 34874603 PMC9787758

[80] Shi H , Koronyo Y , Rentsendorj A , et al. Identification of early pericyte loss and vascular amyloidosis in Alzheimer's disease retina. Acta Neuropathol. 2020;139:813‐836. doi:10.1007/s00401-020-02134-w 32043162 PMC7181564

[81] Mooradian AD , Chung HC , Shah GN . GLUT‐1 expression in the cerebra of patients with Alzheimer's disease. Neurobiol Aging. 1997;18:469‐474. doi:10.1016/s0197-4580(97)00111-5 9390772

[82] Eilam R , Aharoni R , Arnon R , Malach R . Astrocyte morphology is confined by cortical functional boundaries in mammals ranging from mice to human. eLife. 2016;5:5. doi:10.7554/eLife.15915 PMC4945151 27282388

[83] Fan YY , Huo J . A1/A2 astrocytes in central nervous system injuries and diseases: angels or devils? Neurochem Int. 2021;148:105080. doi:10.1016/j.neuint.2021.105080 34048845

[84] Olabarria M , Noristani HN , Verkhratsky A , Rodríguez JJ . Concomitant astroglial atrophy and astrogliosis in a triple transgenic animal model of Alzheimer's disease. Glia. 2010;58:831‐838. doi:10.1002/glia.20967 20140958

[85] Verkhratsky A , Rodrigues JJ , Pivoriunas A , Zorec R , Semyanov A . Astroglial atrophy in Alzheimer's disease. Pflugers Arch. 2019;471:1247‐1261. doi:10.1007/s00424-019-02310-2 31520182

[86] Smit T , Deshayes NAC , Borchelt DR , Kamphuis W , Middeldorp J , Hol EM . Reactive astrocytes as treatment targets in Alzheimer's disease‐systematic review of studies using the APPswePS1dE9 mouse model. Glia. 2021;69:1852‐1881. doi:10.1002/glia.23981 33634529 PMC8247905

[87] Leuzy A , Mattsson‐Carlgren N , Palmqvist S , Janelidze S , Dage JL , Hansson O . Blood‐based biomarkers for Alzheimer's disease. EMBO Mol Med. 2022;14:e14408. doi:10.15252/emmm.202114408 34859598 PMC8749476

[88] Frost GR , Li YM . The role of astrocytes in amyloid production and Alzheimer's disease. Open Biol. 2017;7:7. doi:10.1098/rsob.170228 PMC5746550 29237809

[89] Gomez‐Arboledas A , Davila JC , Sanchez‐Mejias E , et al. Phagocytic clearance of presynaptic dystrophies by reactive astrocytes in Alzheimer's disease. Glia. 2018;66:637‐653. doi:10.1002/glia.23270 29178139 PMC5814816

[90] Sobanov AG . A chart for the evaluation of indices of physical development. Voen Med Zh. 1966;7:87‐89. 5999471

[91] Peron R , Vatanabe IP , Manzine PR , Camins A , Cominetti MR . Alpha‐secretase ADAM10 regulation: insights into Alzheimer's disease treatment. Pharmaceuticals (Basel). 2018;11:11. doi:10.3390/ph11010012 29382156 PMC5874708

[92] Martinez‐Coria H , Green KN , Billings LM , et al. Memantine improves cognition and reduces Alzheimer's‐like neuropathology in transgenic mice. Am J Pathol. 2010;176:870‐880. doi:10.2353/ajpath.2010.090452 20042680 PMC2808092

[93] Hampel H , Vassar R , De Strooper B , et al. The β‐secretase BACE1 in Alzheimer's disease. Biol Psychiatry. 2021;89:745‐756. doi:10.1016/j.biopsych.2020.02.001 32223911 PMC7533042

[94] Korte N , Nortley R , Attwell D . Cerebral blood flow decrease as an early pathological mechanism in Alzheimer's disease. Acta Neuropathol. 2020;140:793‐810. doi:10.1007/s00401-020-02215-w 32865691 PMC7666276

[95] Zhou H , Gao F , Yang X , et al. Endothelial BACE1 impairs cerebral small vessels via tight junctions and eNOS. Circ Res. 2022;130:1321‐1341. doi:10.1161/circresaha.121.320183 35382554

[96] Cheng X , He P , Yao H , Dong Q , Li R , Shen Y . Occludin deficiency with BACE1 elevation in cerebral amyloid angiopathy. Neurology. 2014;82:1707‐1715. doi:10.1212/wnl.0000000000000403 24739782 PMC4032211

[97] Choi JY , Park JH , Jo C , Kim KC , Koh YH . SARS‐CoV‐2 spike S1 subunit protein‐mediated increase of beta‐secretase 1 (BACE1) impairs human brain vessel cells. Biochem Biophys Res Commun. 2022;626:66‐71. doi:10.1016/j.bbrc.2022.07.113 35970046 PMC9349051

[98] Rossner S , Lange‐Dohna C , Zeitschel U , Perez‐Polo JR . Alzheimer's disease beta‐secretase BACE1 is not a neuron‐specific enzyme. J Neurochem. 2005;92:226‐234. doi:10.1111/j.1471-4159.2004.02857.x 15663471

[99] Cho HJ , Kim SK , Jin SM , et al. IFN‐gamma‐induced BACE1 expression is mediated by activation of JAK2 and ERK1/2 signaling pathways and direct binding of STAT1 to BACE1 promoter in astrocytes. Glia. 2007;55:253‐262. doi:10.1002/glia.20451 17091494

[100] Qiao A , Li J , Hu Y , Wang J , Zhao Z . Reduction BACE1 expression via suppressing NF‐κB mediated signaling by Tamibarotene in a mouse model of Alzheimer's disease. IBRO Neurosci Rep. 2021;10:153‐160. doi:10.1016/j.ibneur.2021.02.004 33842919 PMC8019995

[101] Hartlage‐Rübsamen M , Zeitschel U , Apelt J , et al. Astrocytic expression of the Alzheimer's disease beta‐secretase (BACE1) is stimulus‐dependent. Glia. 2003;41:169‐179. doi:10.1002/glia.10178 12509807

[102] McGeer PL , McGeer EG . The amyloid cascade‐inflammatory hypothesis of Alzheimer disease: implications for therapy. Acta Neuropathol. 2013;126:479‐497. doi:10.1007/s00401-013-1177-7 24052108

[103] Liddelow SA , Guttenplan KA , Clarke LE , et al. Neurotoxic reactive astrocytes are induced by activated microglia. Nature. 2017;541:481‐487. doi:10.1038/nature21029 28099414 PMC5404890

[104] Marques F , Sousa JC , Sousa N , Palha JA . Blood‐brain‐barriers in aging and in Alzheimer's disease. Mol Neurodegener. 2013;8:38. doi:10.1186/1750-1326-8-38 24148264 PMC4015275

[105] Zlokovic BV . Neurovascular pathways to neurodegeneration in Alzheimer's disease and other disorders. Nat Rev Neurosci. 2011;12:723‐738. doi:10.1038/nrn3114 22048062 PMC4036520

[106] Winkler EA , Sagare AP , Zlokovic BV . The pericyte: a forgotten cell type with important implications for Alzheimer's disease? Brain Pathol. 2014;24:371‐386. doi:10.1111/bpa.12152 24946075 PMC4423607

[107] Hayes G , Pinto J , Sparks SN , Wang C , Suri S , Bulte DP . Vascular smooth muscle cell dysfunction in neurodegeneration. Front Neurosci. 2022;16:1010164. doi:10.3389/fnins.2022.1010164 36440263 PMC9684644

[108] Xie Y , Kuan AT , Wang W , et al. Astrocyte‐neuron crosstalk through hedgehog signaling mediates cortical synapse development. Cell Rep. 2022;38:110416. doi:10.1016/j.celrep.2022.110416 35196485 PMC8962654

[109] Mulica P , Grünewald A , Pereira SL . Astrocyte‐neuron metabolic crosstalk in neurodegeneration: a mitochondrial perspective. Front Endocrinol (Lausanne). 2021;12:668517. doi:10.3389/fendo.2021.668517 34025580 PMC8138625

[110] Bonvento G , Bolaños JP . Astrocyte‐neuron metabolic cooperation shapes brain activity. Cell Metab. 2021;33:1546‐1564. doi:10.1016/j.cmet.2021.07.006 34348099

[111] Sobol NA , Hoffmann K , Frederiksen KS , et al. Effect of aerobic exercise on physical performance in patients with Alzheimer's disease. Alzheimers Dement. 2016;12:1207‐1215. doi:10.1016/j.jalz.2016.05.004 27344641

[112] De la Rosa A , Olaso‐Gonzalez G , Arc‐Chagnaud C , et al. Physical exercise in the prevention and treatment of Alzheimer's disease. J Sport Health Sci. 2020;9:394‐404. doi:10.1016/j.jshs.2020.01.004 32780691 PMC7498620

[113] Vasconcelos‐Filho FSL , da Rocha Oliveira LC , de Freitas TBC , et al. Neuroprotective mechanisms of chronic physical exercise via reduction of β‐amyloid protein in experimental models of Alzheimer's disease: a systematic review. Life Sci. 2021;275:119372. doi:10.1016/j.lfs.2021.119372 33745893

[114] de Freitas GB , Lourenco MV , De Felice FG . Protective actions of exercise‐related FNDC5/Irisin in memory and Alzheimer's disease. J Neurochem. 2020;155:602‐611. doi:10.1111/jnc.15039 32396989

[115] Chen K , Xu Z , Liu Y , et al. Irisin protects mitochondria function during pulmonary ischemia/reperfusion injury. Sci Transl Med. 2017;9:9. doi:10.1126/scitranslmed.aao6298 PMC5969805 29187642

