# Correction to “Targeting blood brain barrier—Remote ischemic conditioning alleviates cognitive impairment in female APP/PS1 rats”

**DOI:** 10.1111/cns.14788

**Published:** 2024-06-03

Ma Y, Sun W, Bai J, et al. Targeting blood brain barrier—Remote ischemic conditioning alleviates cognitive impairment in female APP/PS1 rats. *CNS Neurosci Ther*. 2024;30:e14613. doi:10.1111/cns.14613

Description of error: Erratum to “Figures 3, 5, 6, 7, and 10.”. We apologize for the careless proofreading. These figures were originally corrected before the proofing stage. However, we now note that the corrected figures were not fully included in the final publication.

Description of error: Corrigendum to “Figures 4 and 11”. We noted that there are minor errors in Figures 4‐b1 and 11‐B, wherein WT group should set to a normalized value of 1.

Please use the following corresponding figures.

**Figure:**
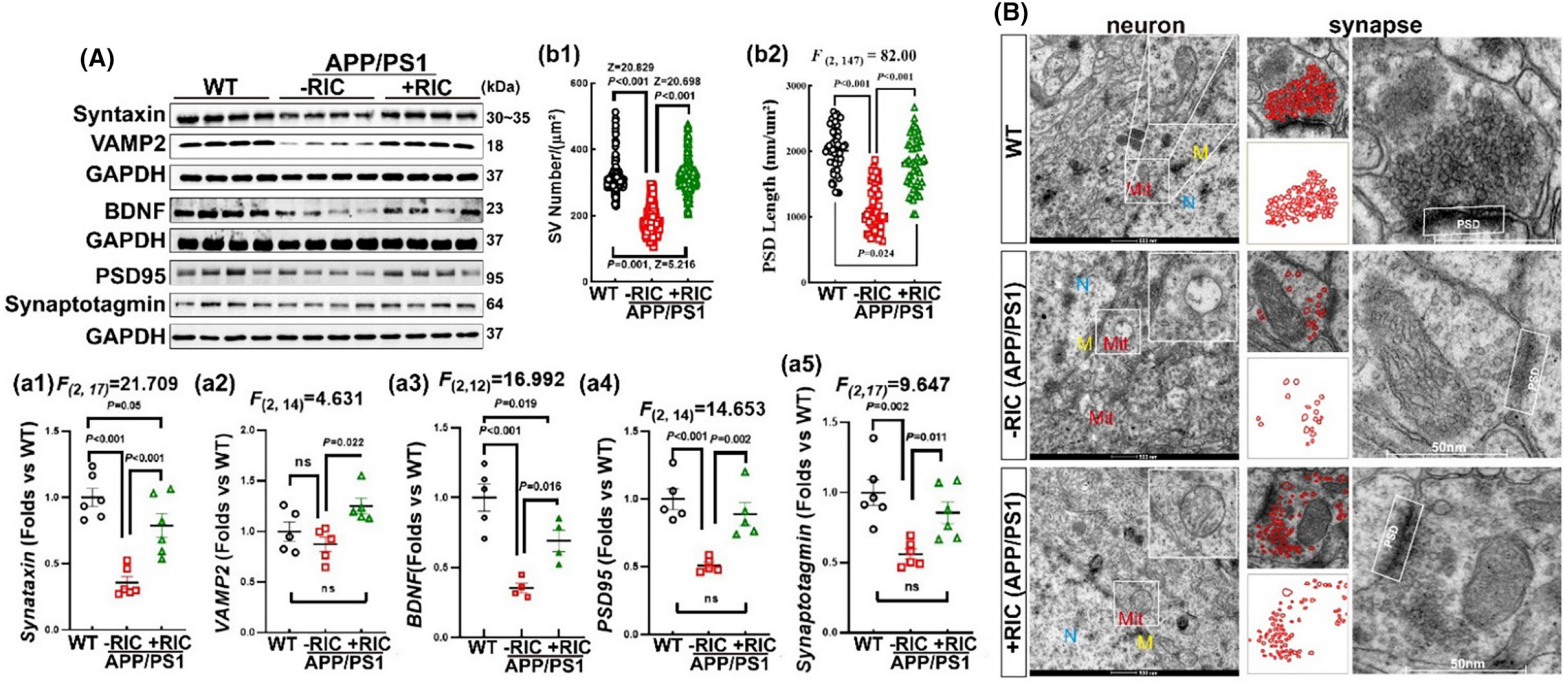
FIGURE 3

**Figure:**
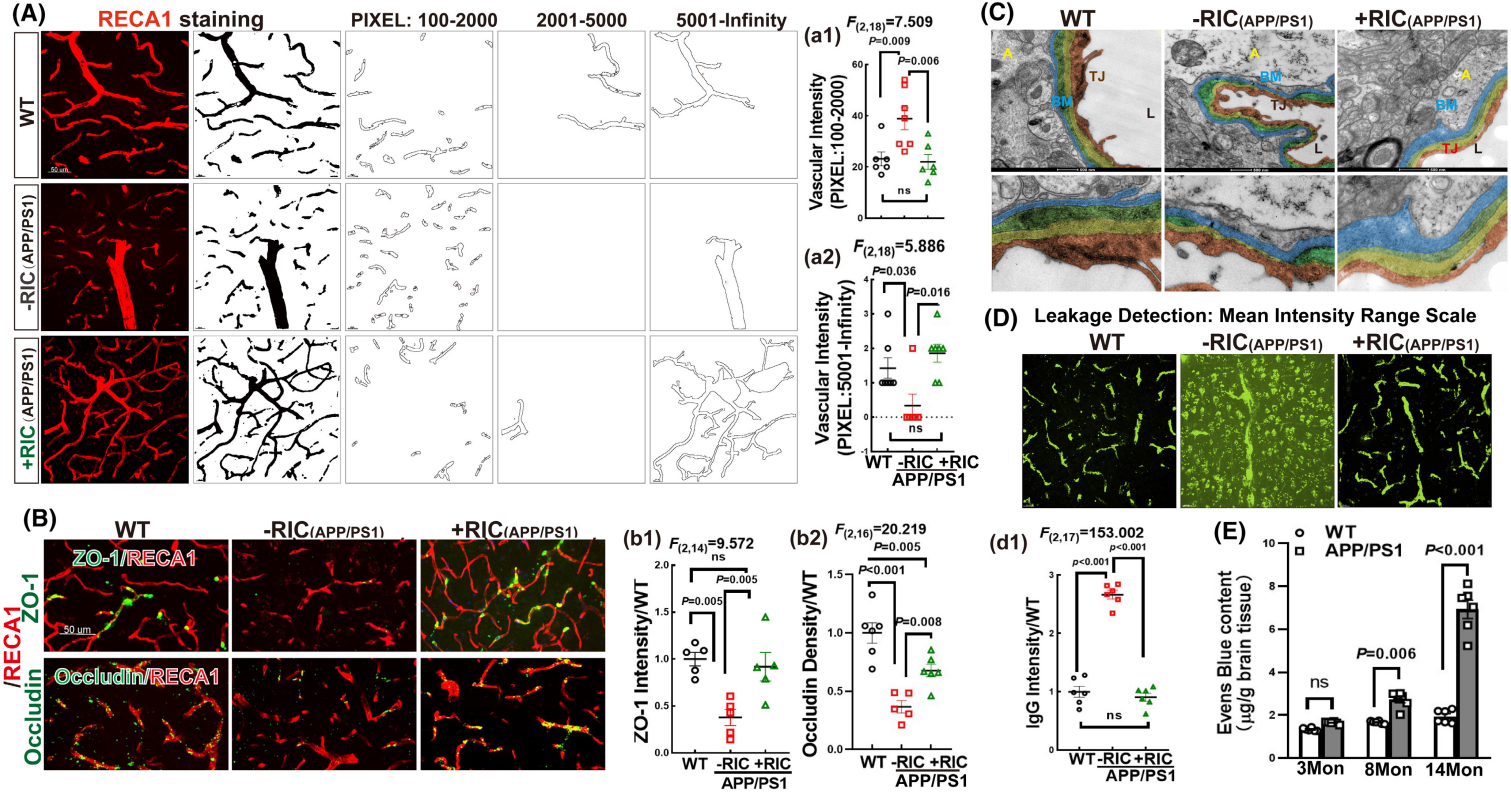
FIGURE 4

**Figure:**
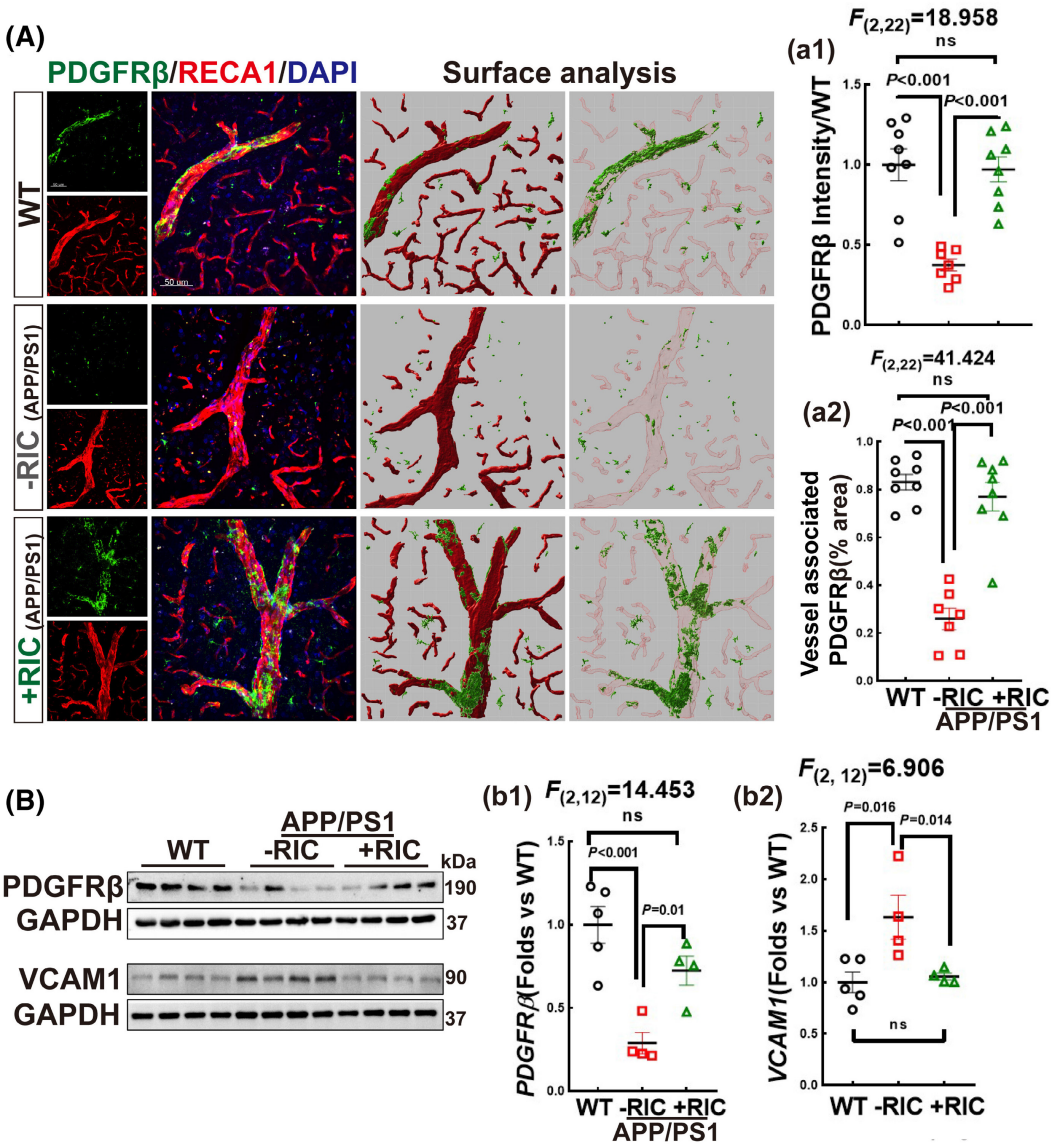
FIGURE 5

**Figure:**
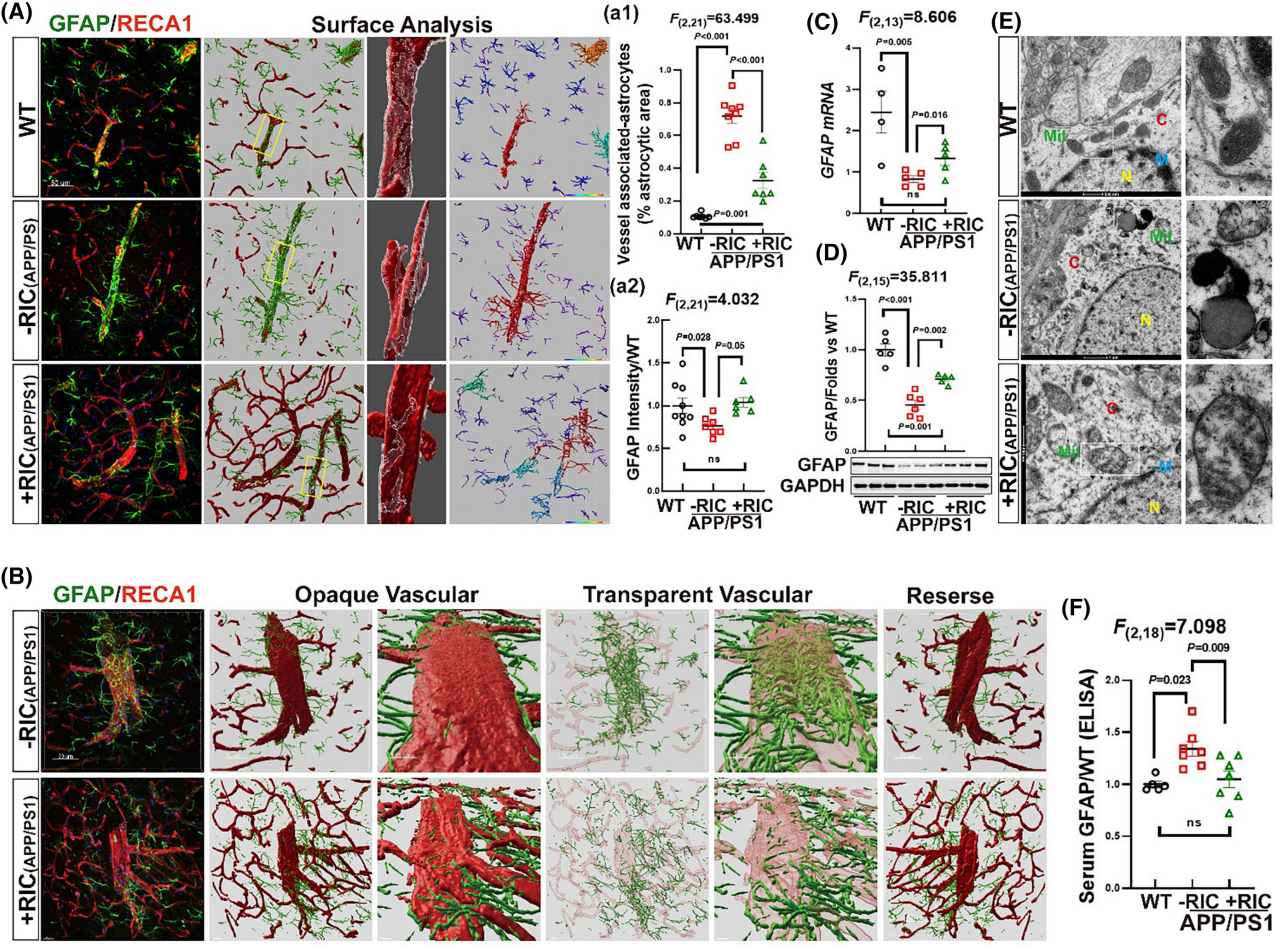
FIGURE 6

**Figure:**
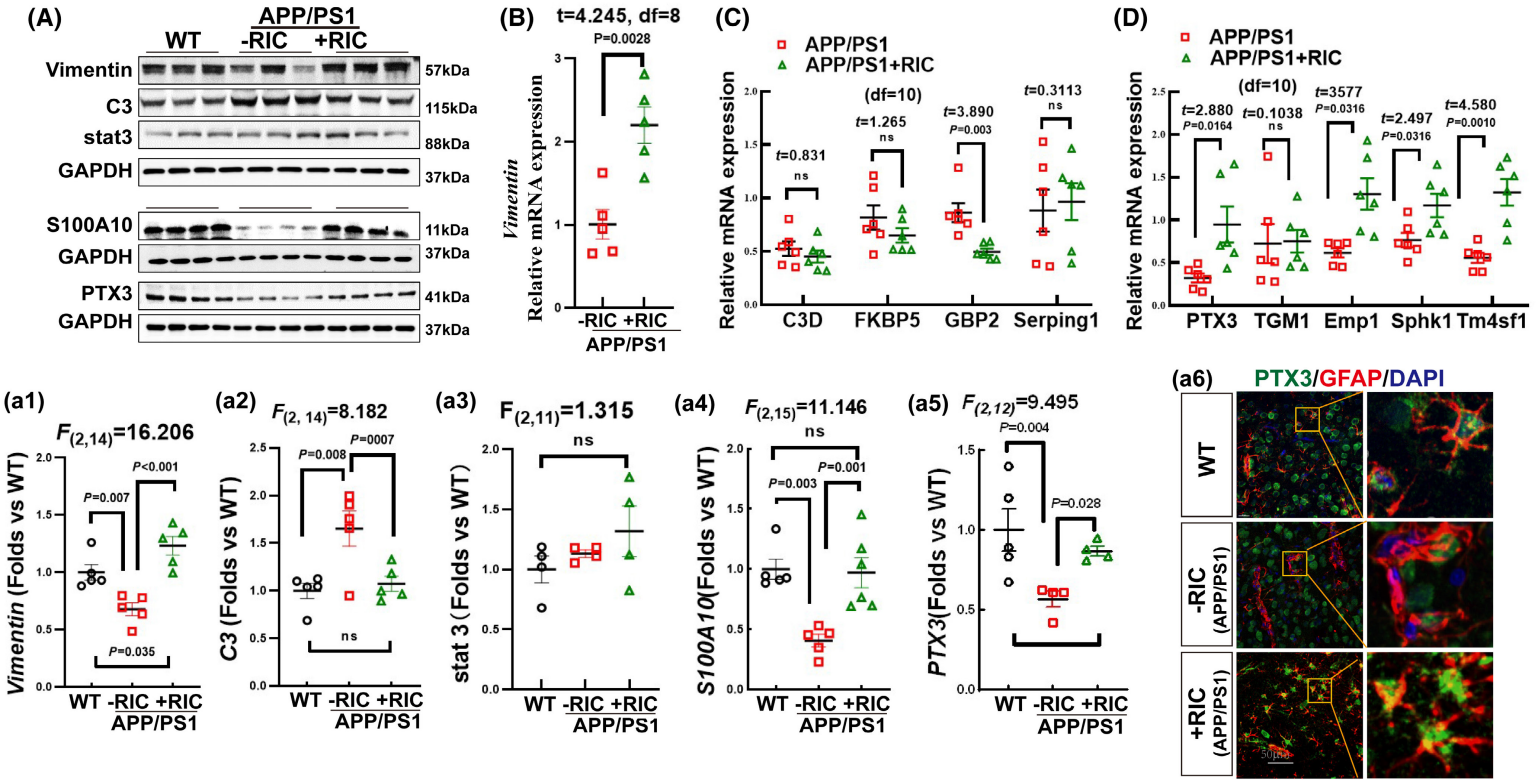
FIGURE 7

**Figure:**
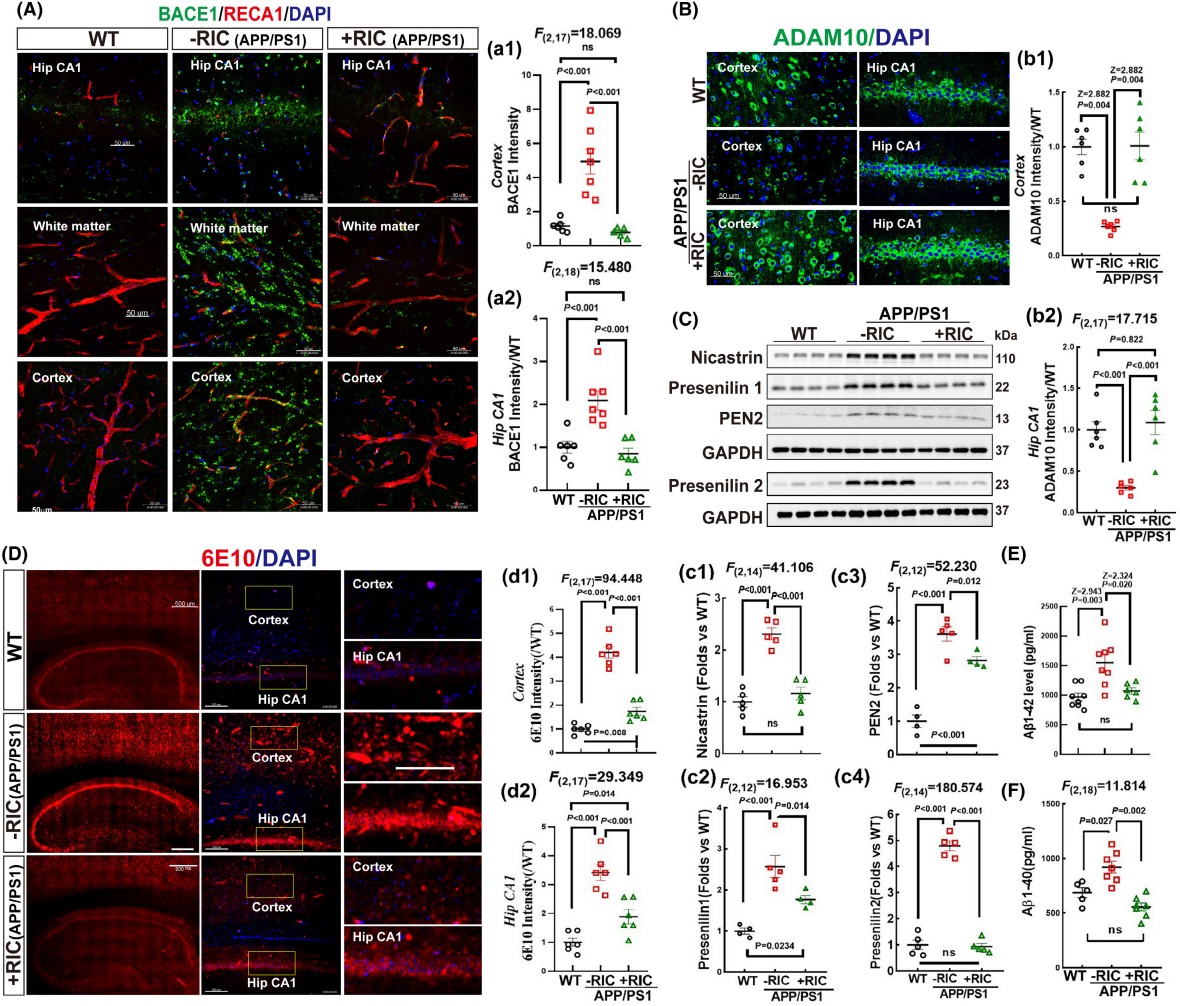
FIGURE 10

**Figure:**
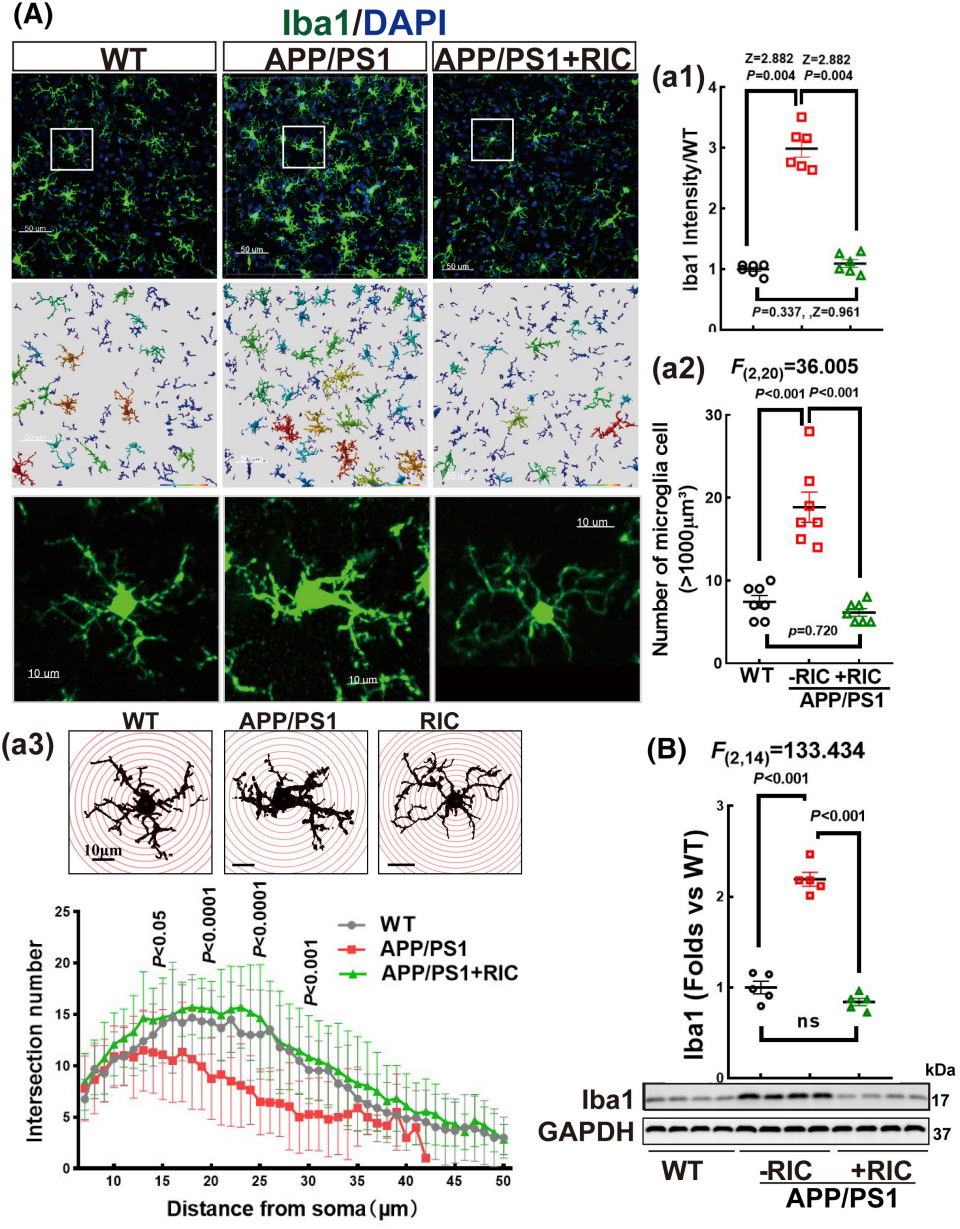
FIGURE 11

