# Supplementary figures and images for: Targeting blood brain barrier—Remote ischemic conditioning alleviates cognitive impairment in female APP/PS1 rats
Source: CNS Neurosci Ther. 2024 Feb 20;30(2):e14613. doi: 10.1111/cns.14613 (PMC10879645; doi:10.1111/cns.14613)

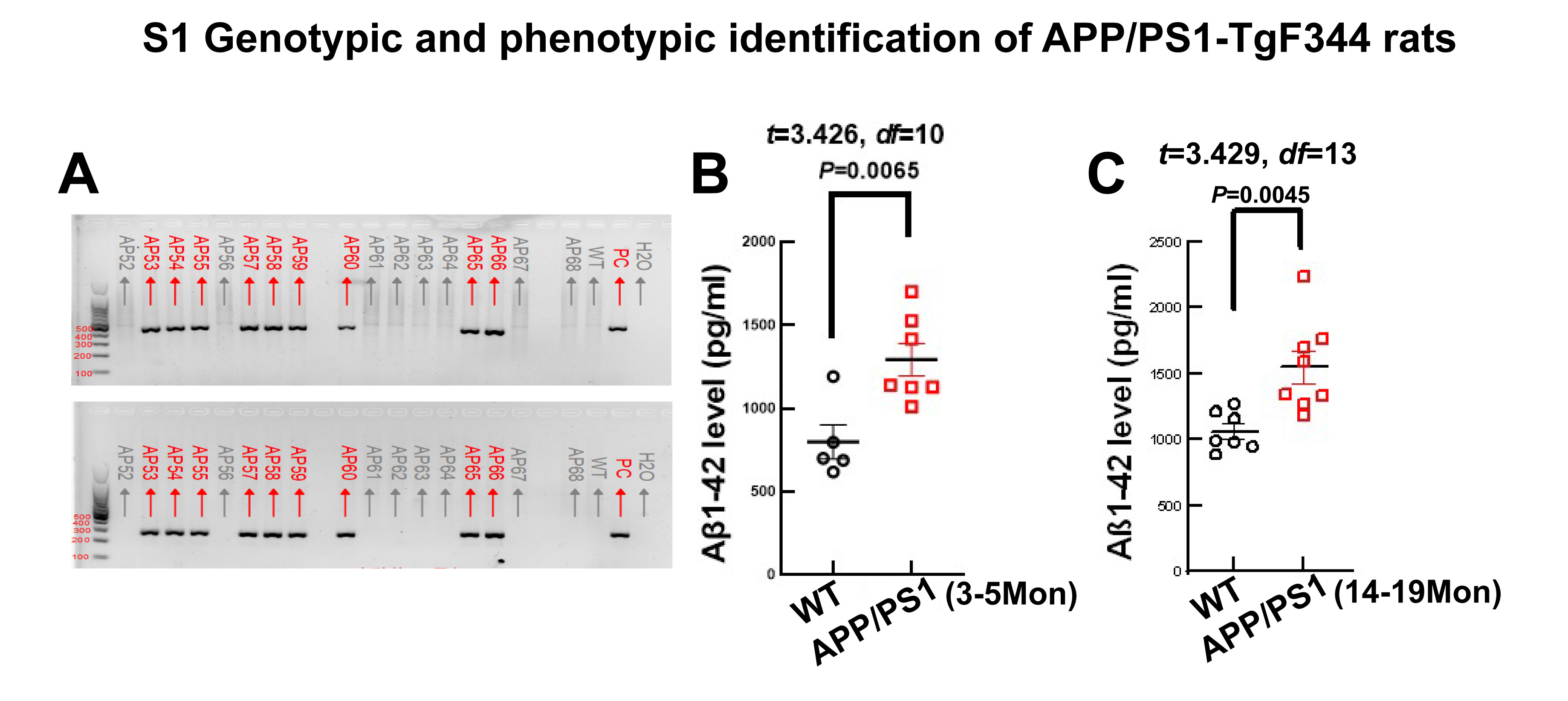

Supplement: Supplementary file 1 — Figure S1. [file CNS-30-e14613-s001.zip › Supplementary Material.jpg]
